# Divergent tissue and sex effects of rapamycin on the proteasome-chaperone network of old mice

**DOI:** 10.3389/fnmol.2014.00083

**Published:** 2014-11-04

**Authors:** Karl A. Rodriguez, Sherry G. Dodds, Randy Strong, Veronica Galvan, Z. D. Sharp, Rochelle Buffenstein

**Affiliations:** ^1^Sam and Ann Barshop Institute for Aging and Longevity Studies, University of Texas Health Science Center at San AntonioSan Antonio, TX, USA; ^2^Department of Physiology, University of Texas Health Science Center at San AntonioSan Antonio, TX, USA; ^3^Department of Molecular Medicine, University of Texas Health Science Center at San AntonioSan Antonio, TX, USA; ^4^Department of Pharmacology, University of Texas Health Science Center at San AntonioSan Antonio, TX, USA

**Keywords:** rapamycin, mTOR, proteasome, heat shock proteins, sexual dimorphic effects, longevity

## Abstract

Rapamycin, an allosteric inhibitor of the mTOR kinase, increases longevity in mice in a sex-specific manner. In contrast to the widely accepted theory that a loss of proteasome activity is detrimental to both life- and healthspan, biochemical studies *in vitro* reveal that rapamycin inhibits 20S proteasome peptidase activity. We tested if this unexpected finding is also evident after chronic rapamycin treatment *in vivo* by measuring peptidase activities for both the 26S and 20S proteasome in liver, fat, and brain tissues of old, male and female mice fed encapsulated chow containing 2.24 mg/kg (14 ppm) rapamycin for 6 months. Further we assessed if rapamycin altered expression of the chaperone proteins known to interact with the proteasome-mediated degradation system (PMDS), heat shock factor 1 (HSF1), and the levels of key mTOR pathway proteins. Rapamycin had little effect on liver proteasome activity in either gender, but increased proteasome activity in female brain lysates and lowered its activity in female fat tissue. Rapamycin-induced changes in molecular chaperone levels were also more substantial in tissues from female animals. Furthermore, mTOR pathway proteins showed more significant changes in female tissues compared to those from males. These data show collectively that there are divergent tissue and sex effects of rapamycin on the proteasome-chaperone network and that these may be linked to the disparate effects of rapamycin on males and females. Further our findings suggest that rapamycin induces indirect regulation of the PMDS/heat-shock response through its modulation of the mTOR pathway rather than via direct interactions between rapamycin and the proteasome.

## Introduction

Rapamycin, an allosteric inhibitor of mechanistic (mammalian) target of rapamycin (mTOR) reportedly increases longevity in mice even when given at an advanced age as an encapsulated formulation in chow. This effect is significantly greater in females (Miller et al., 2014). The biological mechanism and relevance of this sex divergence in response to rapamycin is unclear. Surprisingly, rapamycin has also recently been shown to be an allosteric inhibitor of proteasome activity *in vitro* (Osmulski and Gaczynska, 2013). Further, 20S proteasome activity is reduced in liver tissues of elderly (25 month old) mice treated with rapamycin for 6 months when compared to control animals (Zhang et al., 2014a). However, gene expression studies undertaken in tissues harvested from the same animals showed equivocal effects on proteolytic pathways with differentially expressed genes linked to these pathways both up-regulated and also down-regulated (Fok et al., 2014). Down-regulated proteolytic pathways are at variance with the widely accepted theory that the decline in functionality during aging is linked to an accrual of damaged or misfolded proteins (Ross and Poirier, 2004; David et al., 2010). This breakdown in proteostasis during aging is attributed at least in part to an age-associated decline in the efficiency of the proteolytic machinery (Rodriguez et al., 2010; Grimm et al., 2012). This leads to the concomitant accrual of aggregation-prone cytotoxic proteins that underlie many age-associated pathologies (Bucciantini et al., 2002; Ross and Poirier, 2004).

Age-related changes contributing to compromised proteasome and autophagy function in short-lived species include decreased transcription of some catalytic subunits, altered proteasome subcellular distribution, disruption of lysosomal control, and reduced degradative capacity of both proteolytic machineries (Ferrington et al., 2005; Massey et al., 2006; Rodriguez et al., 2010). In contrast to these declines in proteolytic degradation processes, the proteasome-mediated protein degradation system (PMDS), that includes both ubiquitin-dependent and independent proteasome machinery and associated molecular chaperones, is more robust in naturally long-lived species (Rodriguez et al., 2012; Edrey et al., 2014), long-lived mutants (Kruegel et al., 2011), calorically-restricted animals (Bonelli et al., 2008), and centenarians (Chondrogianni et al., 2000). As such it appears hard to reconcile a decline in proteasome function with rapamycin-induced extended longevity.

The 20S core when doubly capped by 19S regulatory particles is called the 26S proteasome and is primarily responsible for ubiquitinylated protein degradation and the bulk of PMDS related proteolytic activity (Demartino and Gillette, 2007). While 20S proteasomes can exist un-capped *in vivo*, they are mostly inactive and contribute only 20–30% of the total proteasome content in a cell (Babbitt et al., 2005). The 19S regulatory caps through a combination of ATP-dependent (RPT) and ATP-independent (RPN) subunits mediate substrate uptake, by unfolding, deubiquitinylating, and moving proteins through to the 20S core (Demartino and Gillette, 2007). Upstream trafficking of substrates to the proteasome is partially controlled by the heat-shock protein (HSP) family of molecular chaperones. HSP70/72 together with its co-chaperones may have a key regulatory role in PMDS and prolonged efficient function in long-lived species (Grune et al., 2011; Rodriguez et al., 2014). Further, the smaller molecular chaperone, HSP25, has been implicated in the mitigation of protein aggregation in vertebrates (Goldbaum et al., 2009). While chaperone interactions in the PMDS may be critical for maintained protein homeostasis, recently the drug rapamycin has been shown to suppress the heat-shock response in cell culture (Chou et al., 2012), yet nevertheless facilitates prolonged healthspan and extended longevity *in vivo* (Harrison et al., 2009; Miller et al., 2011, 2014; Zhang et al., 2014a).

We question whether this observation of reduced proteasome activity (Osmulski and Gaczynska, 2013; Zhang et al., 2014a) and suppression of the heat-shock response seen *in vitro* (Chou et al., 2012), is observed *in vivo* especially in light of rapamycin-mediated transcriptional regulation of certain proteasome-related genes (Fok et al., 2014). If this is the case, it may reveal that the mechanisms facilitating the increase in healthspan and longevity seen by improved protein homeostasis are independent of rapamycin-induced longevity and healthspan. We test the hypothesis that rapamycin counters the deleterious effects of aging in C57BL/6 mice through differentially suppressing the PMDS. We also ask if there are sex and/or tissue differences in in the various proteasome activities and molecular chaperone responses to rapamycin and whether changes in the mTOR pathway elucidate a mechanism for this rapamycin induced modulation of proteolytic function and lifespan.

## Methods

### Animals

Care of animals followed UT Health Science Center Institutional Animal Care and Use Committees approved procedures. Specific pathogen-free C57BL/6 mice were purchased from the National Institutes of Health colony reared in the Charles River Laboratories at 19 months of age. Mice were maintained under barrier conditions by the UTHSCSA Nathan Shock Center Aging Animal and Longevity Assessment Core and started on the rapamycin/eudragit control diet at ~19 months of age. Six months later at ~25 months of age, the *ad libitum* fed animals were anesthetized with isofluorane, euthanized by cardiac exsanguination and the tissues immediately excised and flash frozen in liquid nitrogen. All the tissues from these animals that were not fasted prior to sacrifice were stored at −80°C until used in analyses.

### Diet preparation

Mice were fed a diet containing either encapsulated rapamycin or empty capsules (eudragit control). Microencapsulated rapamycin or empty microcapsules were incorporated into Purina 5LG6 diet. The rapamycin diet was prepared at 14 ppm using methods described by Harrison et al. (2009) and blood levels checked to confirm appropriate dosing. Data pertaining to the rapamycin blood levels, lifespan, and healthspan effects of these animals have been previously published (Fok et al., 2014; Zhang et al., 2014a).

### Lysates

Mouse tissue (~50–100 mg fat, ~15–30 mg liver, 100–200 mg brain) was cryofractured under liquid nitrogen with a mortar and pestle. Powdered fat was lysed by homogenization in 2× dry weight in Protein Homogenization Buffer (50 mM HEPES, pH 7.6, 150 mM sodium chloride, 20 mM sodium pyrophosphate, 20 mM ß-glycerophosphate, 10 mM sodium fluoride, 2 mM sodium orthovanadate, 2 mM ethylenediaminetetraacetic acid, 1% IGEPAL, 10% glycerol, 1 mM magnesium chloride, 1 mM calcium chloride, 1 mM phenylmethylsulfonyl fluoride, and one tablet/10 ml Complete Mini (EDTA free) protease inhibitor tablets from Roche) for Western blots and in Re-suspension Buffer (RSB) (10 mM HEPES, pH 6.2, 10 mM NaCl, 1.4 mM MgCl_2_) without protease inhibitors at a weight per volume ratio of 1 g of tissue to 2 mL of buffer for peptidolytic assays. The RSB buffer was supplemented with the addition of 1 mM ATP, 0.5 mM DTT, 5 mM MgCl_2_ to help maintain intact 26S subassemblies (Liu et al., 2006). Powdered liver was lysed by homogenization in 5× dry weight in modified RIPA Buffer (150 mM sodium chloride, 50 mM Tris-HCl, pH 7.4, 1 mM ethylenediaminetetraacetic acid, 1 mM phenylmethylsulfonyl flouride, 1% Triton X-100, 1% sodium deoxycholic acid, 0.1% sodium dodecyl sulfate, 1 μM Bortezomib proteasome inhibitor, and one tablet/10 ml protease and phosphatase inhibitor mini tablets (Thermo Fisher Scientific, Waltham, MA, USA) for Western blots. For peptidolytic assays, liver powder was lysed in RSB without protease inhibitors and with ATP supplemented as described above at a weight per volume ration of 1 g of tissue to 5 mL of buffer. Brain tissue was separated and lysed in RSB containing the protease/phosphatase cocktail tablet described above (Thermo Fisher Scientific) for Western blot analyses or RSB without protease inhibitors containing the ATP supplement for peptidolytic assays. Debris was cleared by centrifugation (3000 g for 12 min). The Bio-Rad Protein Assay (Bio-Rad Life Sciences, Hercules, CA) or BCA Assay (Thermo Fisher Scientific) was used to determine protein concentrations.

### Peptidolytic activity

In each assay 20 μg of whole tissue lysates were incubated with 100 μM of substrate specific for the type of proteasome activity. A saturating concentration of proteasome inhibitor N-(benzyloxycarbonyl) leucinyl-leucinyl-leucinal (MG132) was added to parallel samples. The difference of the fluorescence released with and without inhibitor was used as a measure of the net peptidolytic activity of proteasome as previously described using model peptide substrates to represent cleavage after hydrophobic (Chymotrypsin-like; ChTL) residues, basic residues (Trypsin-like; TL) and acidic residues (Post-glutamyl, peptide-hydrolizing; PGPH) (Rodriguez et al., 2010).

### Western blots

Tissue lysates were separated using a 4–12% SDS-PAGE (Biorad Life Sciences) and transferred to nitrocellulose membranes (Biorad Life Sciences). The membranes were probed with antibodies against the following proteasome and chaperone proteins: HSP90 (mouse mAb, SPA831, 1:20K), HSF1 (rabbit pAb, SPA901, 1:5K), HSP70/72 (mouse mAb, SPA810, 1:10K), HSP40 (HDJ1) (rabbit pAb, SPA400, 1:2.5K), HSP25/HSPB1 (rabbit pAb, SPA801, 1:10K), α7 (mouse mAb, PW8110, 1:5K,), RPT5 (mouse mAb, PW8310, 1:5K) (Enzo Life Sciences, Plymouth Meeting, PA, USA) and Carboxyl-terminus of HSP70 Interacting Protein (CHIP) [rabbit mAb(C3B6), #2080, 1:5K] (Cell Signaling Technology, Inc., Danvers, MA, USA). Antibodies against GAPDH (mouse mAb, G8795, 1:30K) (Sigma-Aldrich, St. Louis, MO, USA) were used as a loading control. For mTOR pathway proteins, the following antibodies were used all at 1:1K dilutions: mTOR (rabbit pAb, #2972), phospho-mTOR (Ser2448) (rabbit pAb, #2971), AKT (rabbit pAb, #9272), phospho-AKT (Ser473) (rabbit, pAb, #9271), S6 ribosomal protein (mouse mAb (54D2), #2317), phospho-S6 ribosomal protein (Ser240/244) (rabbit pAb, #2215), 4EBP1 (rabbit pAb, #9452) and phospho-4EBP1 (Thr37/46) (236B4) (rabbit mAb, #2855) (Cell Signaling Technology). Either the GAPDH antibody used above or pan-Actin (mouse mAb, MS-1295-P0, 1:20K) (Thermo-Fisher Scientific) was used as a loading control.

Primary antibodies were detected using anti-mouse IRDye 680LT, or anti-rabbit IR Dye800 CW (Li-Cor, Lincoln, NB, USA) conjugated antibodies. Secondary antibodies were incubated at 1:10K (anti-rabbit) or 1:20K (anti-mouse) for 2 h at room temperature and images were captured and subsequently quantified using the Odyssey Imaging System (Li-Cor) by quantifying fluorescent signals as Integrated Intensities (I.I. K Counts) using the Odyssey Infrared Imaging System, Application Version 3.0 software. We used a local background subtraction method to subtract independent background values from each box, more specifically, the “median” background function with a 3 pixel width border above and below each box was subtracted from individual counts. We calculated ratios for each antibody against the pan-actin or GAPDH loading control using I.I. K Counts. The respective antibody to pan-actin or GAPDH ratio was then used to calculate phosphorylated protein to total protein ratio were applicable.

### Native gel electrophoresis

In this current study, we use an ATP-reconstituting system to maintain the integrity of the 26S proteasome (Liu et al., 2006), however it cannot be ruled out that the fluorogenic proteasome assay measures total proteasome activity for all the proteasome subassemblies, nor does it directly report ubiquitin-dependent activity of the whole 26S. With that caveat, however, we supplement this reliable indirect measure with in-gel assays on native gels.

Proteasome ChTL function was also measured using Native Gel Electrophoresis. This technique enabled us to discriminate if 26S or 20S proteasome activity predominated, the relative quantities of both the double-capped and uncapped proteasome and proteasome specific activities. Fifty micrograms of fractionated lysate from each of the sample groups prepared as described in Subcellular Fractionation above (q.v) were run on a 3–12% non-denaturing, gradient polyacrylamide gel (Life Technologies, Carlsbad, CA). The gel was run at 30 V for 30 min in a 4°C cold cabinet, thereafter the voltage was increased to 35 V for 1 h, 50 V for 1 h and further increased to 75 V for three more hours (Elasser et al., 2005; Tai et al., 2010).

Peptidolytic activity of proteasomes was detected after incubating the gels in a Suc-LLVY-MCA substrate dissolved in 50 mM Tris pH 8.0, 5 mM MgCl_2_, 1 mM DTT, 2 mM ATP, and 0.02% SDS for 15, 30, and 60 min at 37°C. Proteasome bands were identified by the release of highly fluorescent, free AMC (Elasser et al., 2005; Vernace et al., 2007; Rodriguez et al., 2012). Fluorescence was quantitated using ImageJ software (http://imagej.nih.gov/ij/). Following the in-gel assay, the protein from the gel was transferred to nitrocellulose using the i-blot transfer apparatus (Life Technologies) and subjected to Western blotting analyses using the α7 antibody described above to determine where the 26S and 20S proteasome complexes lay. The IRDye conjugating antibodies and the Odyssey Imaging System (Li-Cor) were used to quantitate the amounts of α7 signal (See Western Blots above).

### Statistical analyses

Prism 5 (GraphPad Software, Inc.) was used to analyze and graph the mTOR pathway Western blot data. An unpaired two-tailed *t*-test or Mann Whitney test was used to obtain *p*-values. *P*-values below 0.05 were considered significant. Proteasome and chaperone data was analyzed with Sigma Plot (v.11) using Two-Way ANOVA. Statistical significance was set at the *p* < 0.05 level with Tukey and Holm-Sidak corrections to counteract the probability of false positives. Cluster Analysis was performed using Cluster 3.0 and Treeview v 1.16r2 to generate output (open source code http://bonsai.hgc.jp/~mdehoon/software/cluster/; University of Tokyo, 2002). Data were log transformed to normalize the activity and protein expression data, and the cluster was generated using the group medians and hierarchical clustering with an uncentered Pearson Correlation to generate a complete linkage to create the most unbiased set of clusters (Yeung and Ruzzo, 2001; De Hoon, 2002).

## Results

### Rapamycin-treated females showed increases in both chymotrypsin-like and trypsin-like 26S proteasome activity in brain, but not in liver or visceral fat

**Figure 1:**
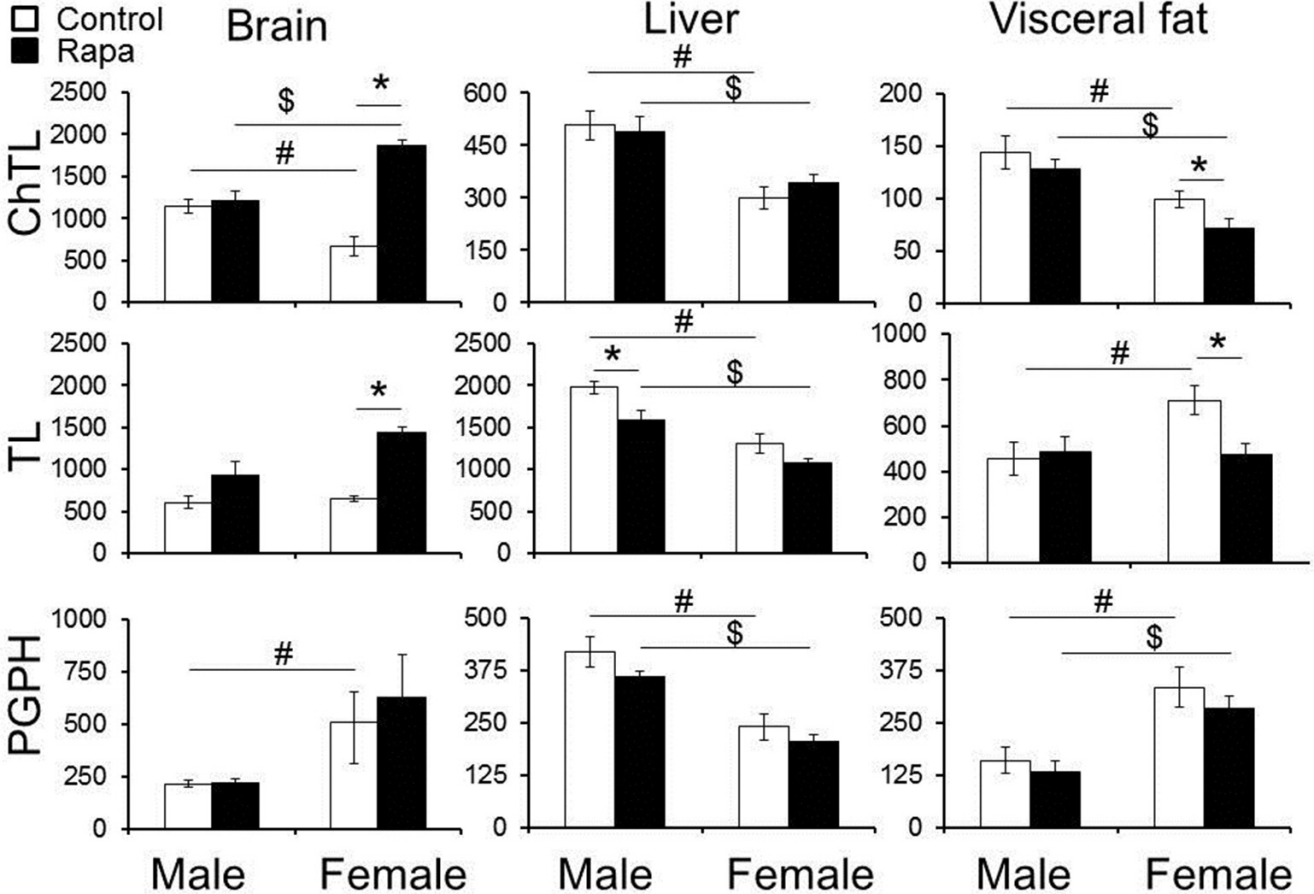
**Changes in chymotrypsin-like (ChTL) and trypsin-like (TL) 26S proteasome activity in rapamycin-treated lysates from old animals depended upon the tissue type and sex of the animals tested**. Significant increase was observed for both ChTL (3X) and TL (2X) 26S proteasome activities in whole brain lysates from female animals only. No significant effects were evident in male brains. Liver TL activity declined (0.8X) with rapamycin treatment in males. Rapamycin treatment led to a decline in visceral fat ChTL (0.7X) and TL (0.7X) activity in females but not in males. PGPH activity did not show any significant differences between treated or untreated animals in any of the tissues examined. The y-axis indicates net proteasome activity in pmol/min/μg lysate for each of the respective activities measured. Statistically significant differences (Two-Way ANOVA, *p* < 0.05) comparing treatment vs. vehicle (∗) and/or male vs. female untreated (#) or male vs. female treated ($) are indicated (*n* = 5 brain, fat, *n* = 6 liver).

Peptidolytic activity of the proteasome was measured in old (25 month) male and female, rapamycin-treated and untreated, brain, liver, and visceral fat lysates using model peptide substrates specific for each of the three types of catalytic sites. The core particle contains three cleavage sites that degrade polypeptides or unfolded proteins severing peptide bonds on the carboxyl side of hydrophobic (“chymotrypsin-like”; ChTL), basic (“trypsin-like”; TL), or acidic (“peptidylglutamyl peptide hydrolyzing” or “caspase-like”; PGPH) residues (Demartino and Gillette, 2007). To determine net proteasome activity, assays were run in parallel with and without the proteasome inhibitor MG132. Divergent responses were evident with regards to treatment, sex, and peptidolytic activities (Figure 1; summarized in Table 1). In untreated animals, males showed significantly higher ChTL activity in brain (*p* = 0.01), liver (*p* = 0.002), and fat (*p* = 0.03) (Figure 1, top panels) than observed in females. TL activity, while still higher for males in liver (*p* = 0.009), was the same in brain and higher in females visceral fat (*p* = 0.02). PGPH activity was higher in lysates from female animals compared to males in both brain (*p* = 0.03) and fat (*p* = 0.01), but in liver, PGPH activity was higher in untreated males compared to females (*p* = 0.003) (Figure 1, bottom panels).

**Table 1:**
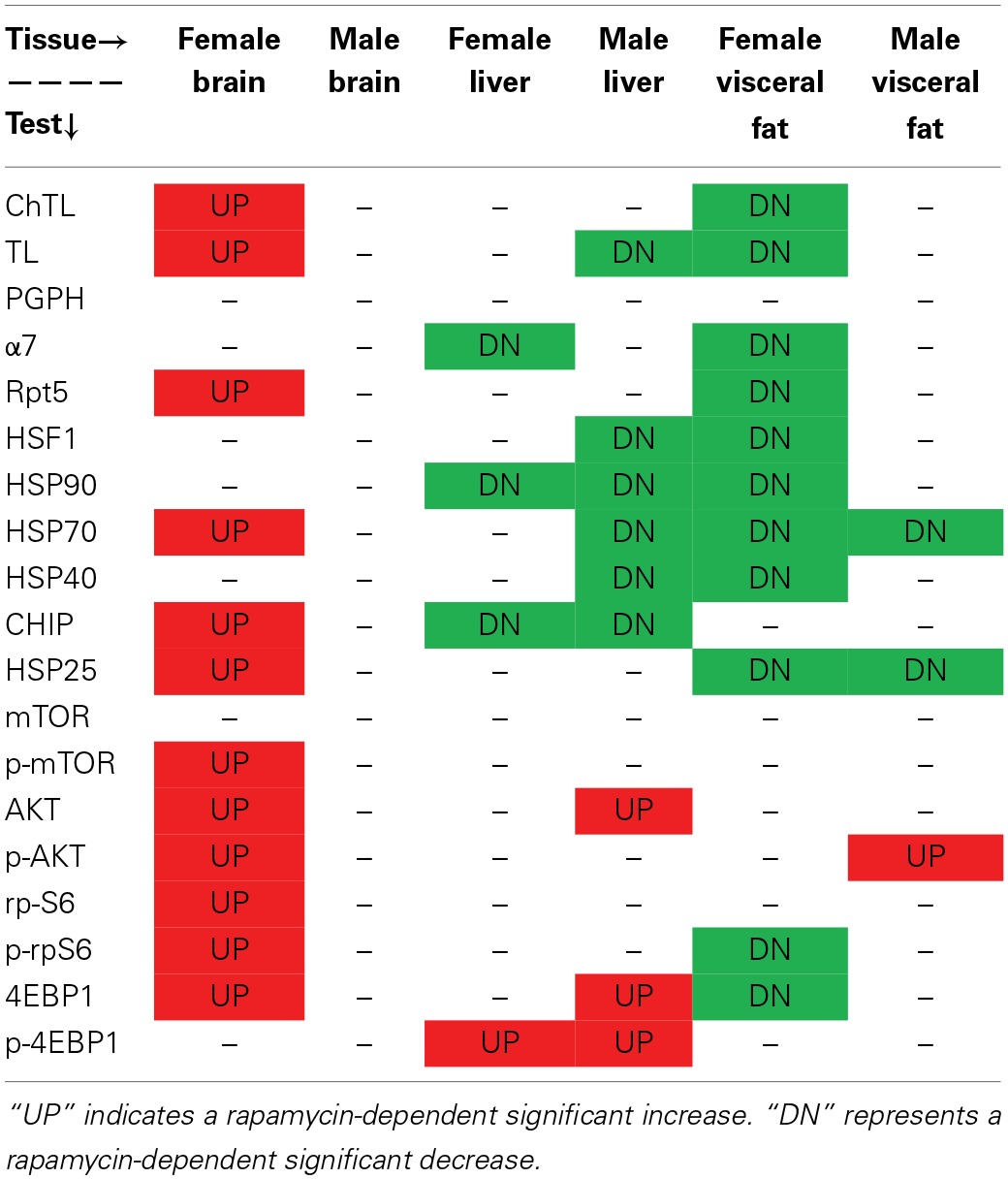
**Rapamycin-influenced changes in markers of proteasome, chaperone, and mTOR pathway in old male and female brain, liver, and visceral fat lysates**.

“UP” indicates a rapamycin-dependent significant increase. “DN” represents a rapamycin-dependent significant decrease.

Rapamycin effects were also tissue-specific but in general proteasome activity in females was more responsive to rapamycin treatment. In whole brain tissue lysates males showed no change in proteasome activity, whereas in females, the change in proteasome activity was significant, being nearly 3-fold higher for ChTL (*p* = 0.0009) and 2-fold higher for TL (*p* = 0.0005) substrates in treated compared to untreated samples (Figure 1, top left). In the liver lysates only male TL activity changed significantly, with a 20% decline in activity in the rapamycin treated (*p* = 0.02) (Figure 1, middle). Finally, proteasome activity from visceral fat lysates showed significant declines in ChTL (*p* = 0.05) and TL (*p* = 0.01) activities of 30% in samples from treated females compared to controls. Activity in male fat lysates did not change with treatment (Figure 1, right panels). Rapamycin treatment had no effect on PGPH activity in either sex or in any of the tissues tested (Figure 1, left panels). Interestingly, ChTL activity showed the greatest sexual dimorphism with sex-differences evident in the activities of samples from both rapamycin-treated and untreated controls. Activity was higher in male control samples, whereas females showed the higher activity in rapamcyin treated samples (Figure 1, top left).

**Figure 2:**
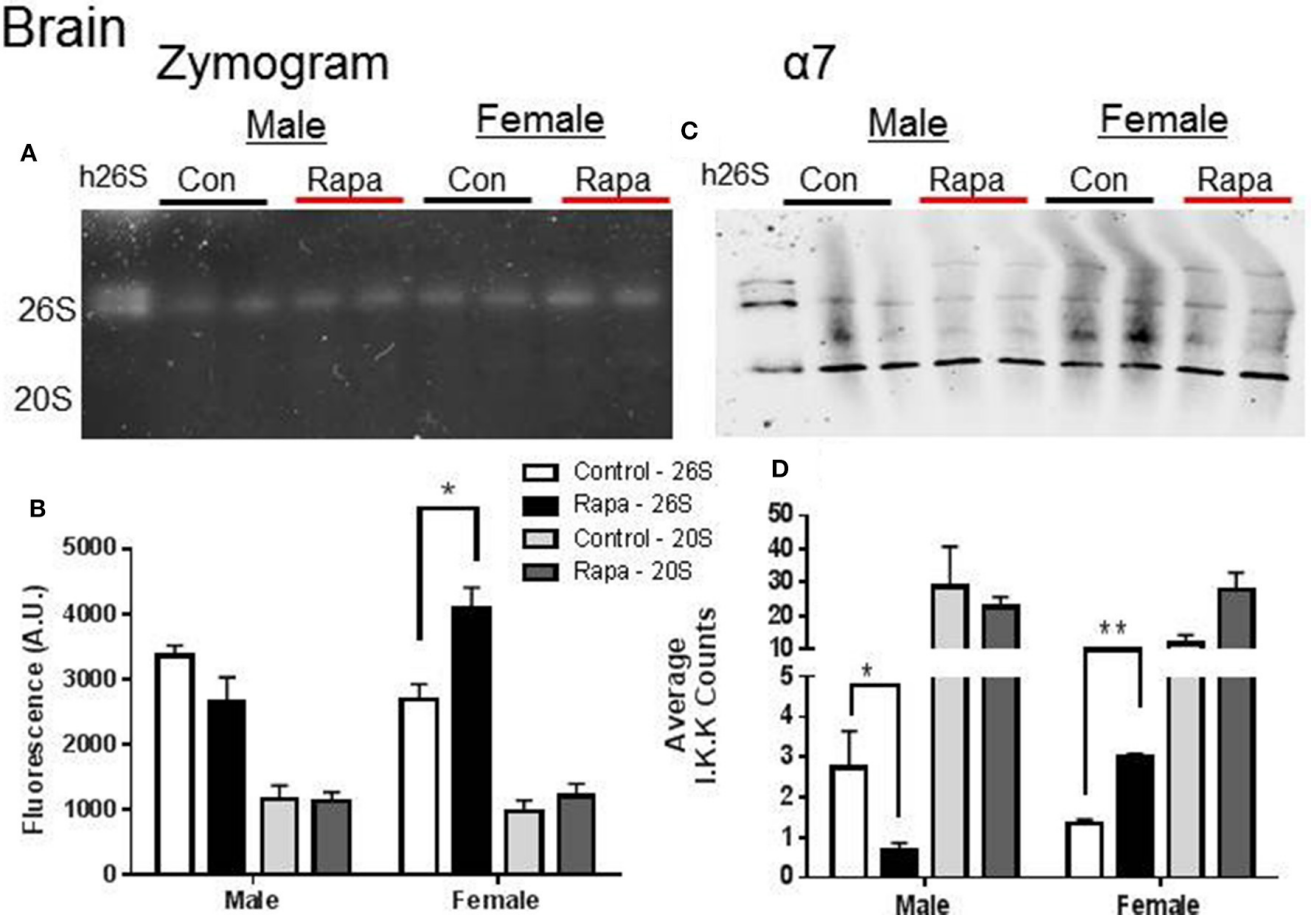
**Female Brain Lysates showed a rapamycin-dependent increase in 26S proteasome. (A)** Representative zymogram of Chymotrypsin-like (ChTL) proteasome activity after native gel electrophoresis of 50 μg of brain lysate from control and rapamycin-treated old male and female animals. **(B)** Quantitation of zymogram showed that there was a treatement-induced increase in 26S-specific ChTL proteasome activity in female brain lysates (*n* = 4, ^*^p < 0.05 Two-Way ANOVA). **(C)** Representative immunoblot of α7 proteasome subunit after transfer from native gel. **(D)** Similarly, α7 subunit showed a 26S-specific change (increase in females, decrease in males; ^*^p < 0.05, ^**^p < 0.01; *n* = 4, Two-Way ANOVA) in assembled proteasome content. It did not seem that change in male proteasome content was sufficient to influence a change in activity.

**Figure 3:**
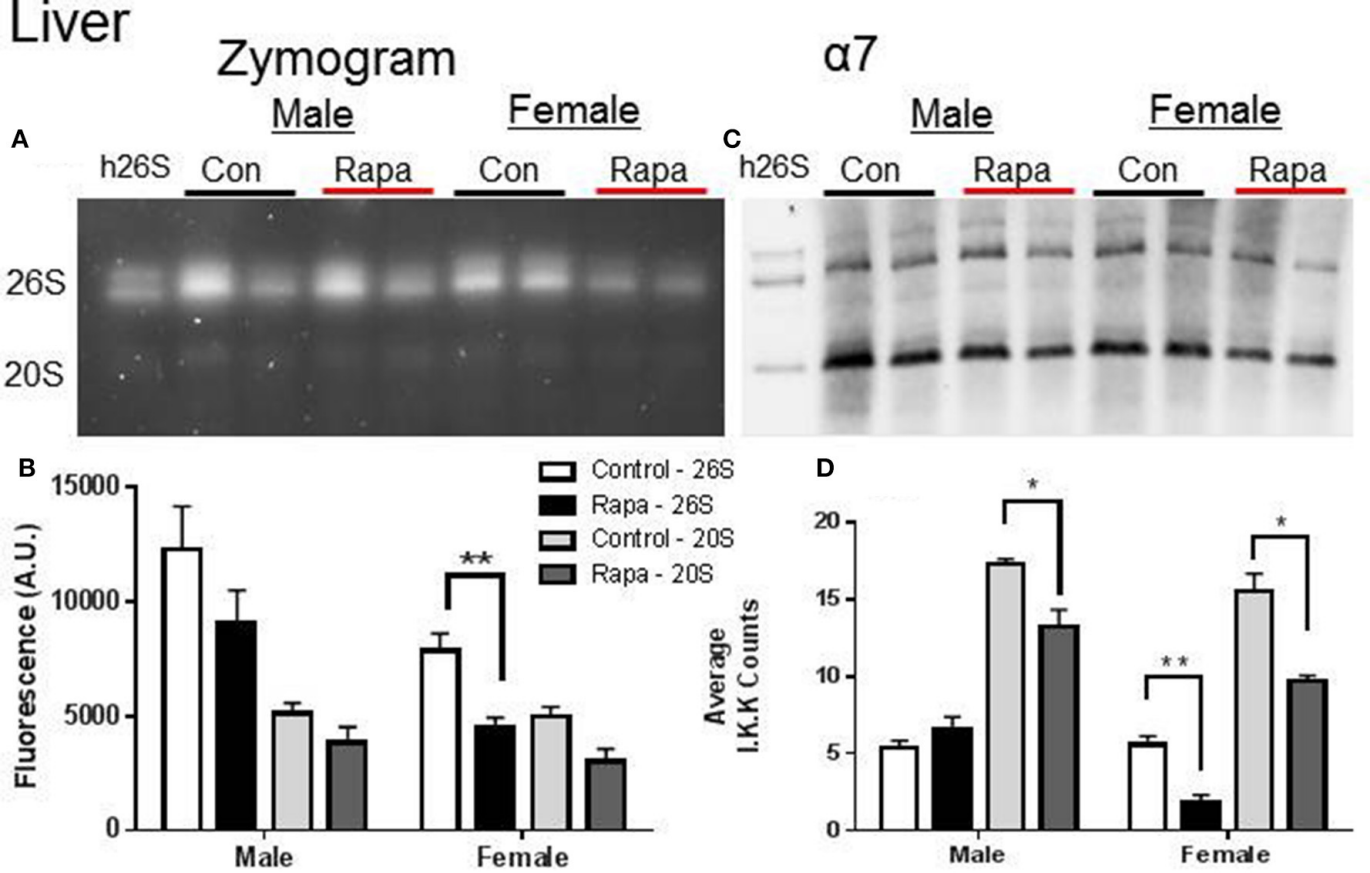
**Liver Lysates revealed potential rapamycin-dependent declines in proteasome activity. (A)** Representative zymogram of Chymotrypsin-like (ChTL) proteasome activity after native gel electrophoresis of 50 μg of liver lysate from control and rapamycin-treated old male and female animals. **(B)** Quantitation of zymogram showed that there was a treatement-induced decrease in 26S-specific ChTL proteasome activity in female brain lysates (*n* = 4, ^*^p < 0.05 Two-Way ANOVA). **(C)** Representative immunoblot of α7 proteasome subunit after transfer from native gel. **(D)** The calculated amounts of the α7 subunit showed significant decreases at both the 26S (females) and 20S sites (males and females) (^*^p < 0.05, ^**^p < 0.01; *n* = 4, Two-Way ANOVA).

**Figure 4:**
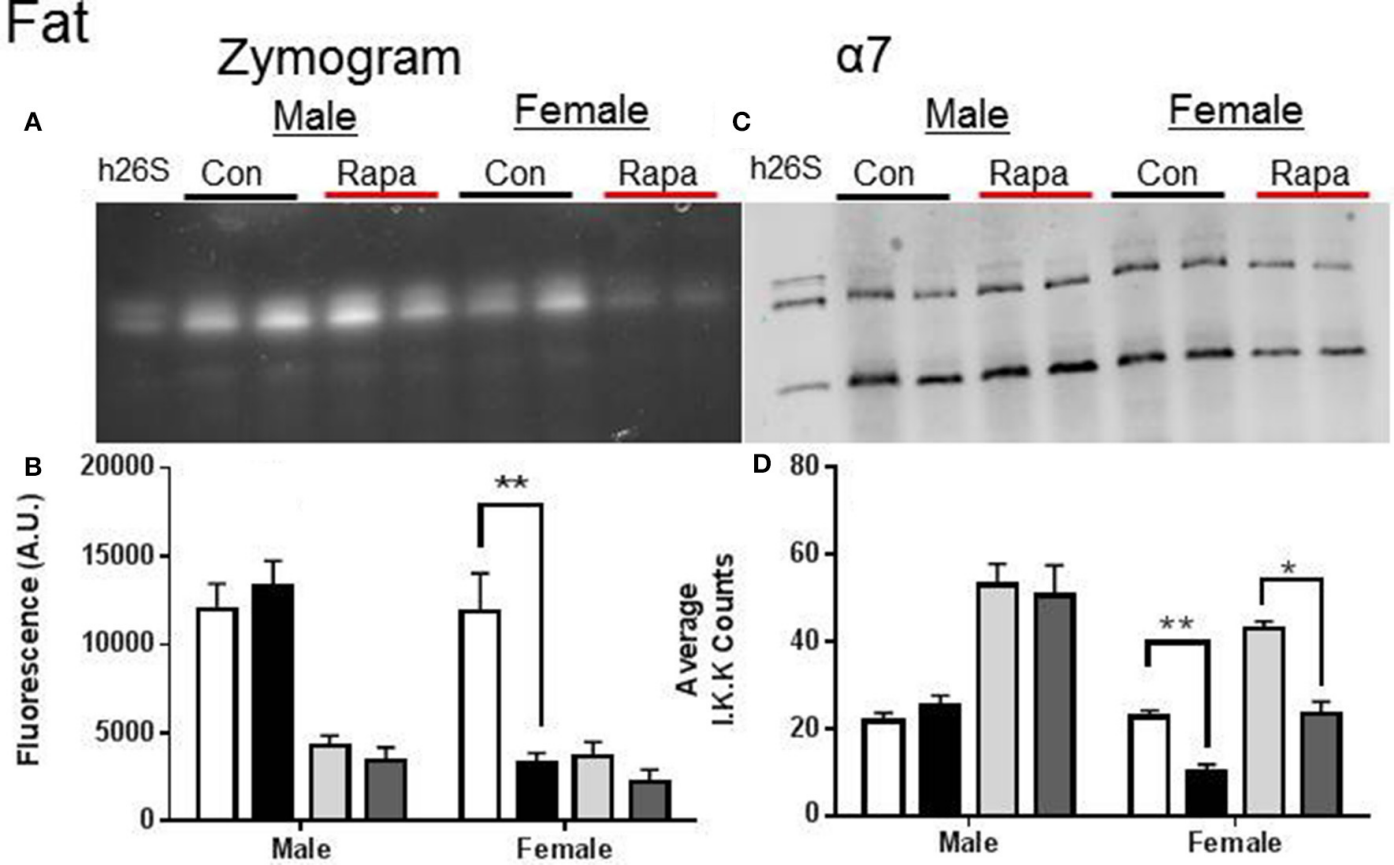
**Female Fat Lysates also showed rapamycin-influenced declines in proteasome activity. (A)** Representative zymogram of Chymotrypsin-like (ChTL) proteasome activity after native gel electrophoresis of 50 μg of visceral fat lysate from control and rapamycin-treated old male and female animals. **(B)** Quantitation of the zymogram revealed that there was a treatment-dependent decrease in female 26S ChTL activity (*n* = 4, ^*^p < 0.05 Two-Way ANOVA). **(C)** Representative immunoblot of α7 proteasome subunit after transfer from native gel. **(D)** The α7 subunit quantitation indicated significant decreases in both female 26S and 20S content (^*^p < 0.05, ^**^p < 0.01; *n* = 4, Two-Way ANOVA).

Native gel electrophoresis has been used to determine if the proteasome remains intact in a higher molecular weight form (i.e., 26S) or exists disassembled (20S) (Elasser et al., 2005; Rodriguez et al., 2012). We used this technique to determine if either the 20S or 26S proteasome assembly, was affected by rapamycin treatment or showed sex-specific differences (Figures 2–4). In tissue lysates from female mouse brains, the in-gel assay measuring ChTL activity (Figures 2A,B) showed a significant increase in 26S proteasome activity in a rapamycin-dependent manner (*p* = 0.02), but not in 20S activity. In contrast ChTL activity in male mouse brains did not change (*p* > 0.05). The immunblot analyses of α7 (Figures 2C,D) revealed that the 26S proteasome content of this protein significantly increased in brain lysates of rapamycin treated female mice (*p* = 0.003). Interestingly, the proteasome α7 decreased significantly in rapamycin-treated male mice brain samples compared to those of the male control group (Figures 2C,D; *p* = 0.02), though this did not reflect on activity. 20S proteasome content did not change for either sex.

Zymograms for 26S proteasome activity showed a significant decline in both liver (Figures 3A,B) and fat (Figures 4A,B) tissue lysates of rapamycin-treated females (*p* = 0.008 brain, *p* = 0.009 fat) with no change in proteasome activity for either sex at the 20S site. A significant decline in α7 protein content occurred in 26S proteasomes measured in liver samples from rapamycin-treated females (*p* = 0.004). Further, 20S α7 protein content declined in both male (*p* = 0.02) and female liver samples (*p* = 0.001) (Figures 3C,D). Proteasome content in fat tissue lysates (α7) also decreased significantly for both the 26S (*p* = 0.003) and 20S (*p* = 0.04) sites of treated female sample whereas α7 content did not change in the fat samples from males.

### Levels of proteasome-related chaperones changed more in treated females than in treated males

**Figure 5:**
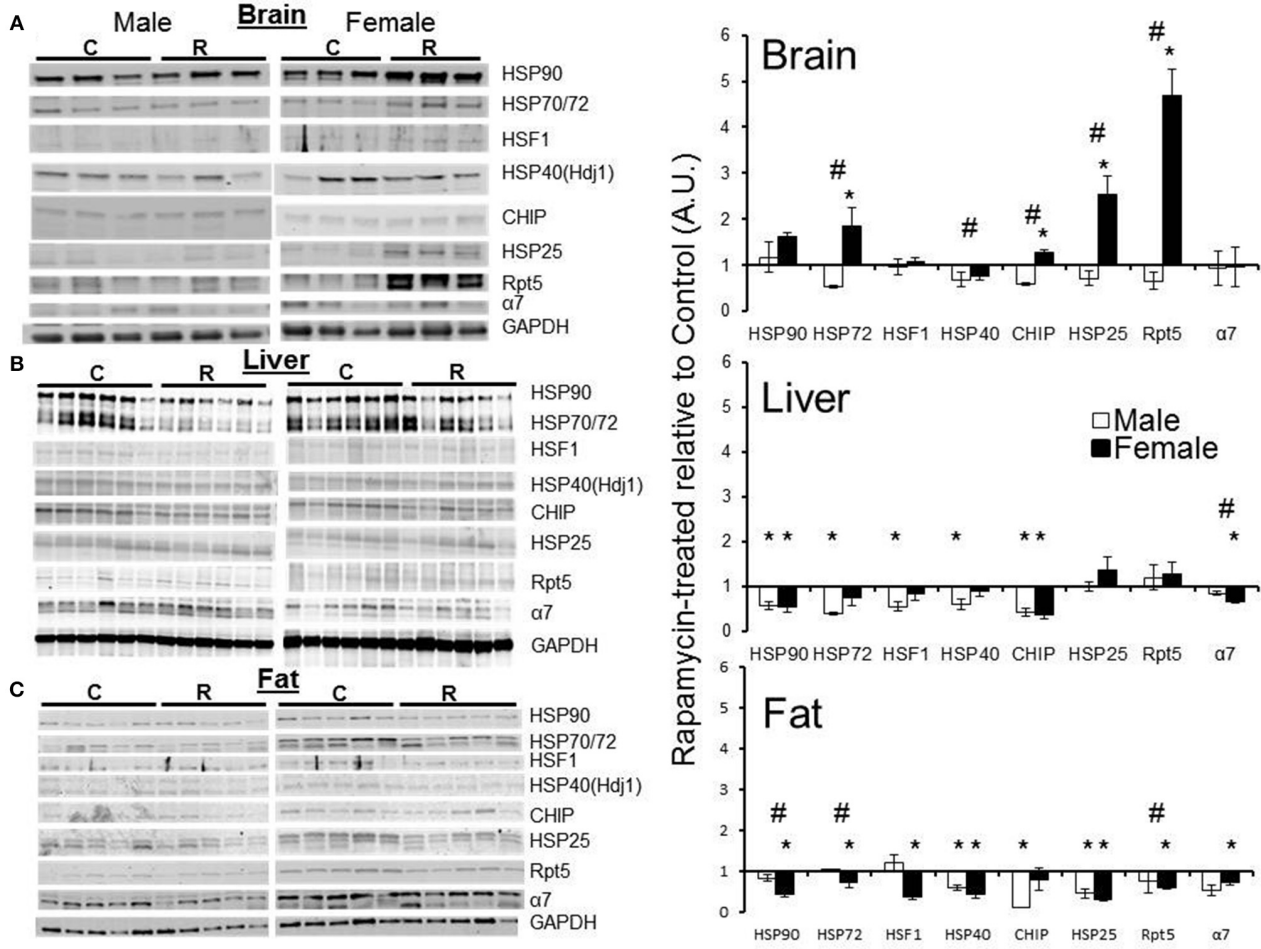
**Both proteasome subunits and proteasome-related chaperones in brain, liver, and visceral fat lysates revealed tissue and sex dependent treatment-related changes**. Changes in protein expression as measured by Western blots relative to GAPDH, and normalized to old control in brain lysates from old (25 mo) male and female rapamycin-fed mice **(A)** indicated a female-specific, treatment-related increase for HSP72, HSP40, CHIP, HSP25, and RPT5. All these proteins also showed sex-dependent differences as well. In liver lysates **(B)** both sexes showed a treatment-related decline in all proteins tested except for HSP25 and RPT5. There was only a significant sex difference in α7. In visceral fat lysates **(C)** females again showed a treatment-related change most strikingly for the proteasome markers α7 and RPT5. Visceral fat showed significant sex-dependent differences in all proteins tested except for HSP25. Significant differences between male and female pairs (*n* = 3 brain, *n* = 5 fat, *n* = 6 liver; Two-Way ANOVA, *p* < 0.05) are indicated by (∗) treatment-related change or (#) sex-dependent change.

Western blot analyses using a panel of antibodies representing major chaperone families and key proteasome subunits was undertaken in the tissues harvested from rapamycin-fed and Eudragit-(vehicle) fed controls. Tissues from three male and three female 25 month old mice were analyzed. In lysates from male samples, rapamycin had no effect on chaperone levels (Figure 5A). In female brain lysates, the levels of HSP70, CHIP, and HSP25 proteins increased in treated samples compared to controls (Figure 5A). Further, the 19S cap protein RPT5 also showed a higher protein content in these brain lysates from rapamycin-treated females (Figure 5A). Proteins were normalized to GAPDH whose mRNA is eIF4E insensitive and would not change with rapamycin treatment (Livingstone et al., 2010), These data are indicative of an increased 26S population and RPT5 levels correlated with an increase in ChTL and TL proteasome activity in brain lysates (Figure 1). In liver, with the exception of HSP25 (which did not change), there was a decline in heat shock proteins in both sexes with treatment (Figure 5B). While α7 levels also decreased in males and females, this did not correlate with any loss in proteasome activity (Figures 1, 5B). In visceral fat, HSF1 as well as HSP90, HSP70, HSP40, and HSP25 decreased significantly in samples from treated female animals (Figure 5C). Both RPT5 and α7 also showed protein declines in fat samples from rapamycin-treated females that correlated with a decline in ChTL and TL proteasome activity. Interestingly, samples from treated males showed declines in CHIP, HSP, and HSP25 (Figure 5C). Lastly, HSP90, HSP70, and RPT5 showed significant differences between males and females sample in their immuno-detection (Figure 5C).

### mTOR pathway markers responded to rapamycin treatment in females but these changes were also tissue dependent

**Figure 6:**
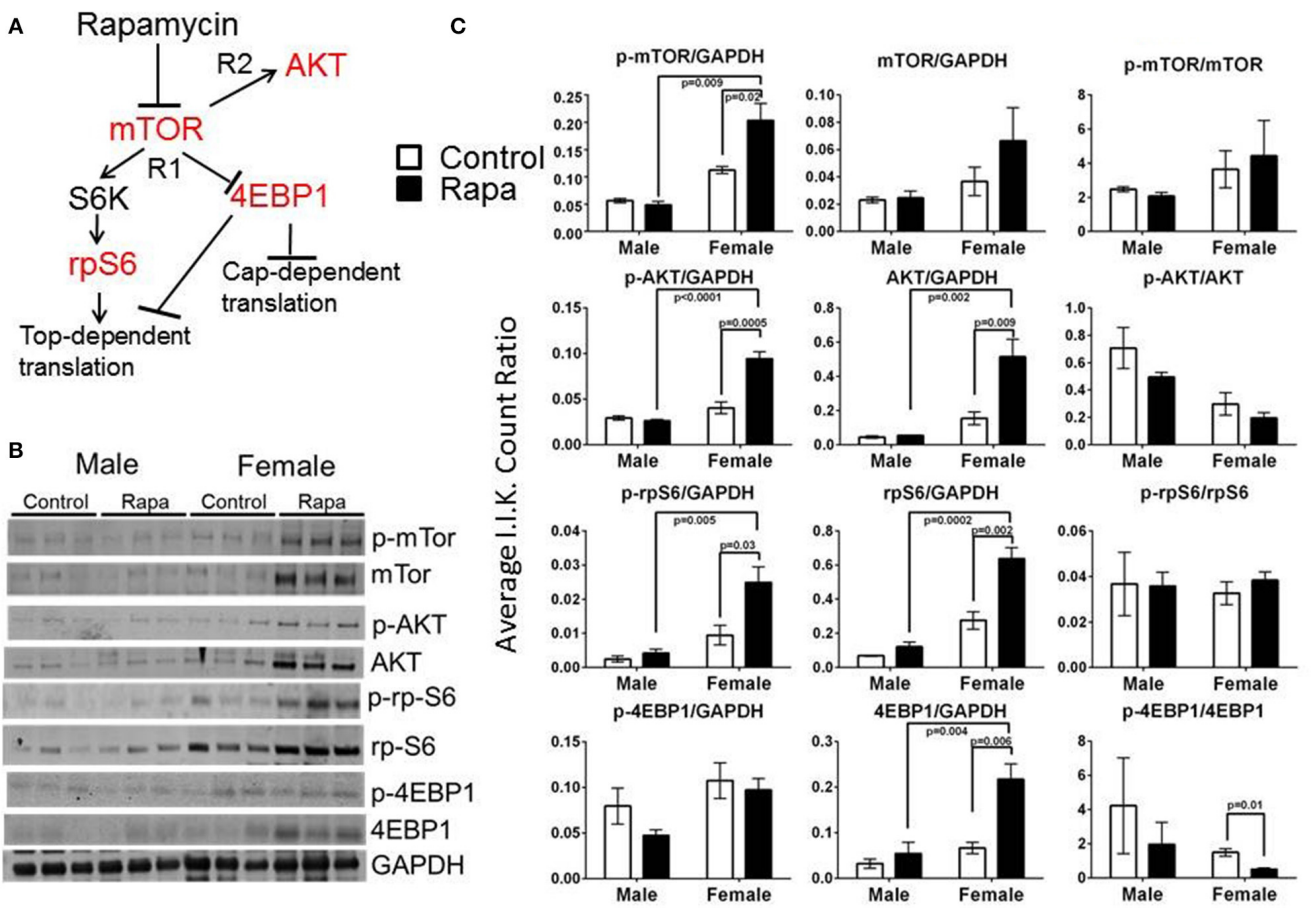
**Treatment with rapamycin altered expression of key proteins of the mTOR pathway in female brain lysates. (A)** Basic schematic of the relationship of the proteins tested (highlighted in red) to mTOR (R1 indicates mTORC1-dependent branch while R2 indicates the mTORC2-dependent branch). **(B)** Changes in protein expression of these markers of the mTOR pathway measured in brain lysates from 25 month-old male and female mice (rapamycin-fed vs. control) and quantified Integrated Intensities (I.I. K Counts) relative to GAPDH (I.I. K Count Ratio) **(C)** showed that rapamycin influenced change in mTOR itself and both proteins upstream of mTOR (AKT) as well as its downstream targets (rpS6 and 4EBP1). Both total and phosphorylated (p) forms are presented as well as the ratio of the phosphorylated vs. the total protein. Significant *p*-values as derived by Two-Way ANOVA (*n* = 3) using Prism GraphPad Software is indicated for each set of proteins.

To examine for a correlation between the changes in proteasome activity and chaperone profile with the mTOR pathway, we immunoassayed a subset of mTOR pathway proteins and their phosphorylated forms (p) in brain, liver, and visceral fat lysates from old male and female, rapamycin-fed animals and compared to similarly aged controls relative to Actin (also insensitive to rapamycin) or GAPDH (Livingstone et al., 2010) (Figure 6B). Female rapamycin-treated brains had higher levels of p-mTOR (Ser2448), and both total and phosphorylated AKT (Ser473) and rpS6 (Ser240/244). We also observed an increase in total 4EBP1 and unchanged p-4EBP1 (Thr36/47) levels which led to significant decline in the ratio of p-4EBP1/4EBP1 (Figure 6C). In male lysates there was no significant difference in the phosphorylated or total protein expression of these four mTOR pathway proteins, with rapamycin-treatment (Figures 6B,C).

**Figure 7:**
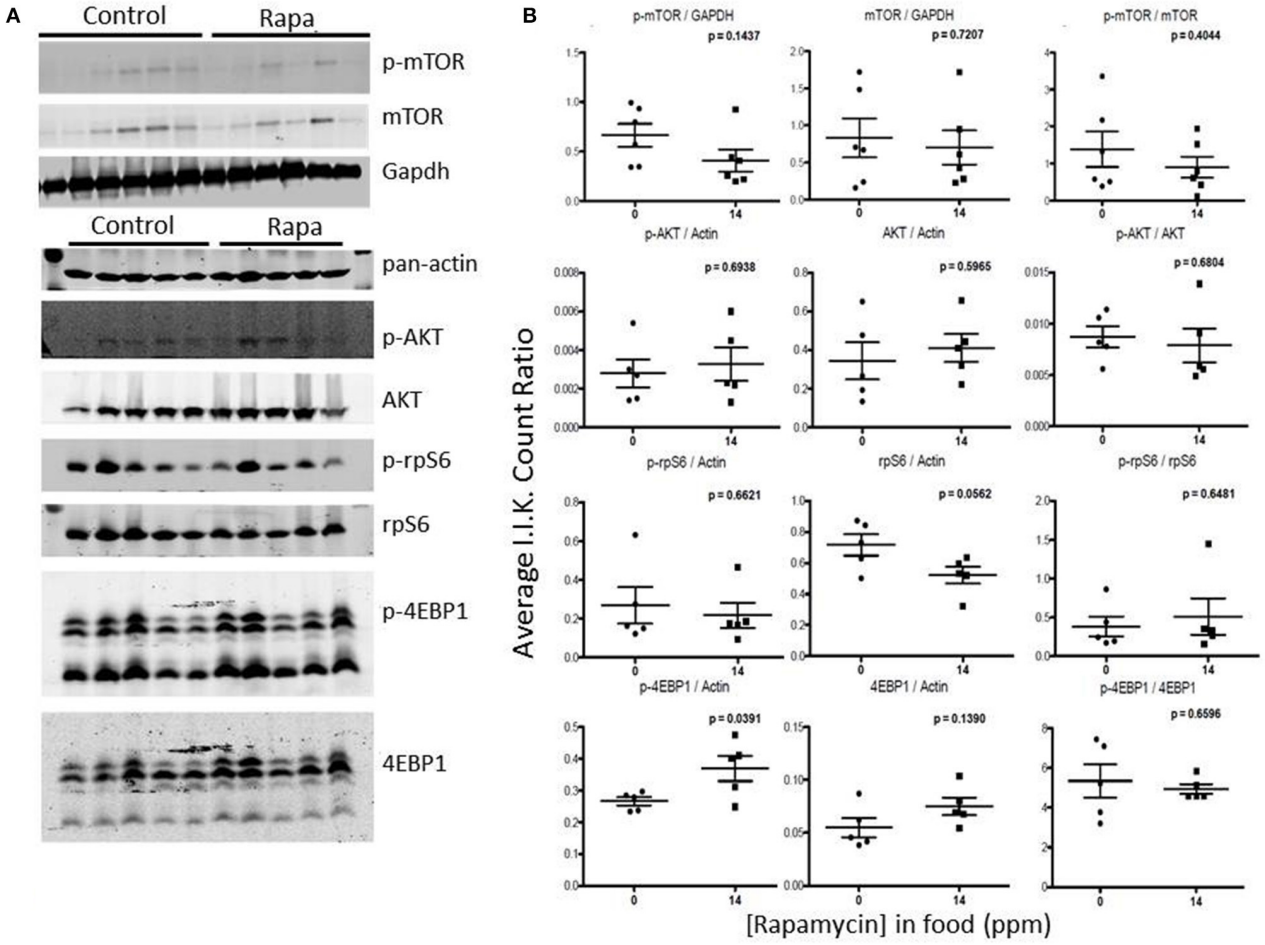
**Liver lysates from female C57BL6 mice did not show a clear pattern of change in mTOR signaling. (A)** Liver lysates from 25 month female rapamycin-treated and control animals were analyzed by Western blot for several mTOR-pathway related proteins and quantified using I.I. K Counts relative to pan-Actin or GAPDH **(B)**. *P*-values (unpaired *t*-test, Prism GraphPad) are indicated above each dot plot (*n* = 6).

**Figure 8:**
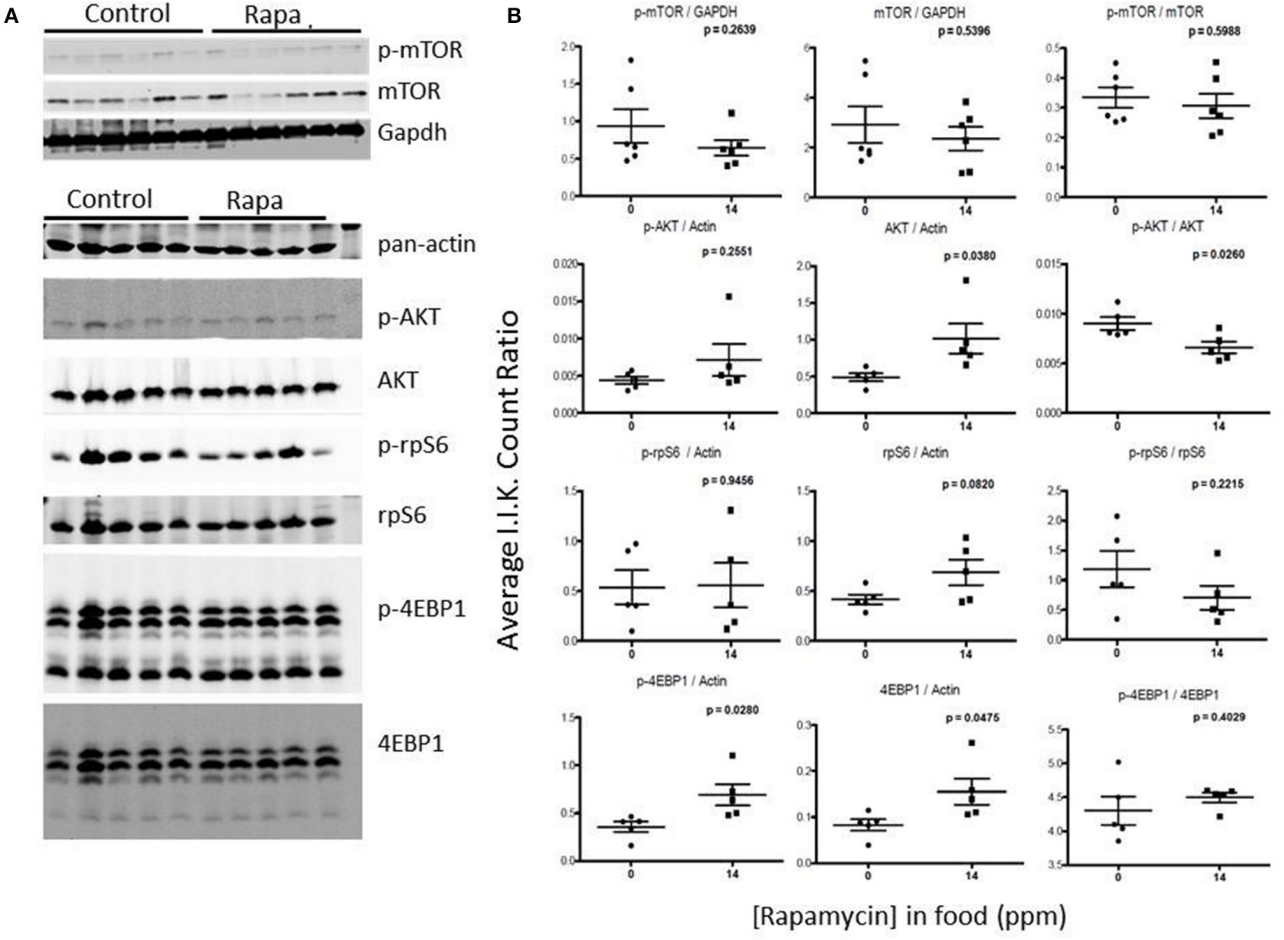
**Liver lysates from male C57BL6 mice also did not show a clear pattern of change in mTOR signaling. (A)** Western blots from lysates from 25 month-old males and quantitation using I.I. K Counts relative to pan-Actin or GAPDH **(B)**. *P*-values (unpaired *t*-test, Prism GraphPad) are indicated above each dot plot (*n* = 6).

Rapamycin had little effect on protein expression of mTOR related proteins in liver tissue (Figures 7, 8). Only p-4EBP1 (Thr36/47) in liver lysates of females was significantly higher with rapamycin (Figures 7A,B) whereas in liver lysates from males, rapamycin induced increased expression of both p-4EBP1 (Thr36/47) and total 4EBP1 (Figures 8A,B) as well as total AKT. The latter change led to a lower ratio of p-AKT (Ser473) to total AKT (Figure 8B).

**Figure 9:**
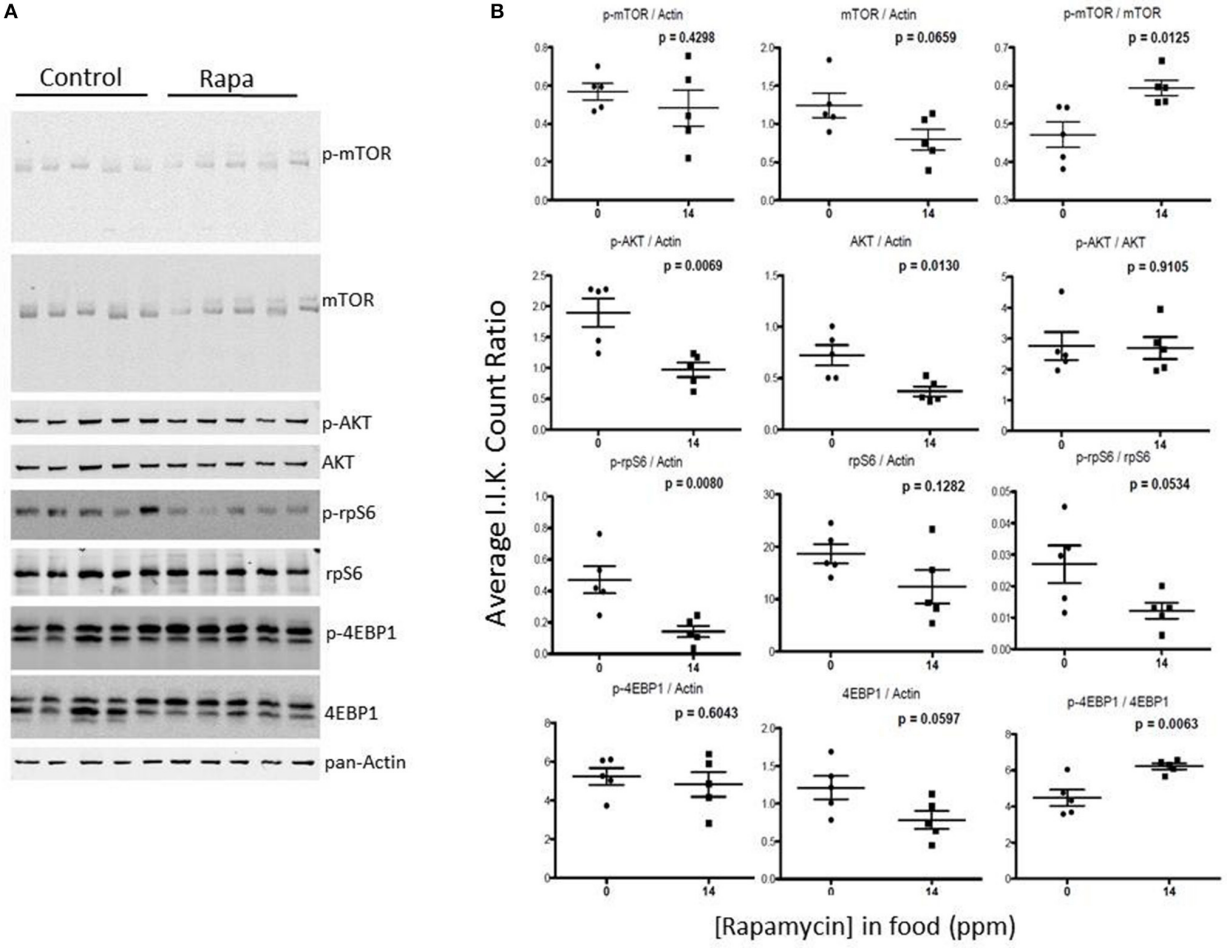
**The phosphorylation state of downstream mTOR pathway markers changed significantly in visceral fat lysates from rapamycin-treated females**. Measuring protein content of several mTOR pathway markers analyzed from visceral fat lysates taken from 25 month rapamycin-fed and control female mice via immunoblot **(A)** quantified using I.I. K Counts relative to pan-Actin **(B)** revealed a decrease in p-rp-S6 which also lowered the ratio (phosphorylation state) of p-rpS6 to total rp-S6. Total 4EBP1 also declined, increasing the phosphorylation state. *P*-values, derived using Prism GraphPad Software, are indicated for each dot plot (*n* = 5).

**Figure 10:**
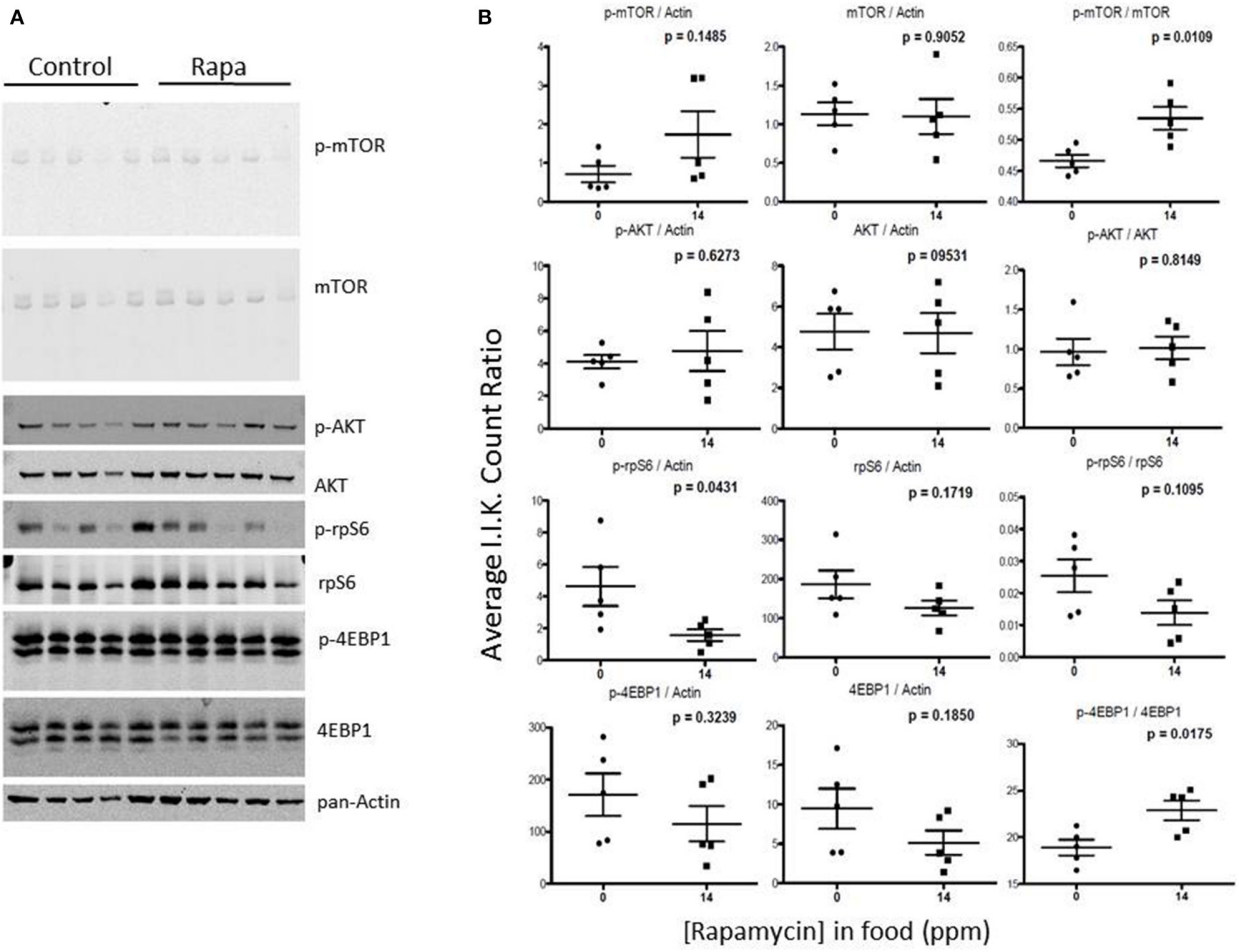
**mTOR pathway proteins changed minimally in visceral fat lysates from rapamycin-treated males**. Lysates created from the visceral fat of 25 month-old rapamycin-fed and control male mice were analyzed via Western blot **(A)** and quantified using I.I. K Counts **(B)** relative to pan-Actin as a loading control. Only phosphorylated AKT showed a significant change. *P*-values (unpaired *t*-test, Prism GraphPad) are shown above each dot plot (*n* = 5).

Visceral fat lysates from rapamycin-treated females showed a trend toward a decrease in p-AKT (Ser473), total mTOR and downstream effectors p-rp-S6 (Ser240/244) and total 4EBP1 (Figures 9A,B). These rapamycin mediated declines in protein expression led to altered ratios of the phosphorylated form to the total proteins (Figure 7B). The effects of rapamycin on the expression of these proteins were attenuated in males. Only phosphorylated AKT (Ser473) and the ratios of p-mTOR (Ser2448) to total mTOR significantly increased (Figures 10A,B).

### Cluster analyses revealed that brain proteasome activity is most influenced by rapamycin

**Figure 11:**
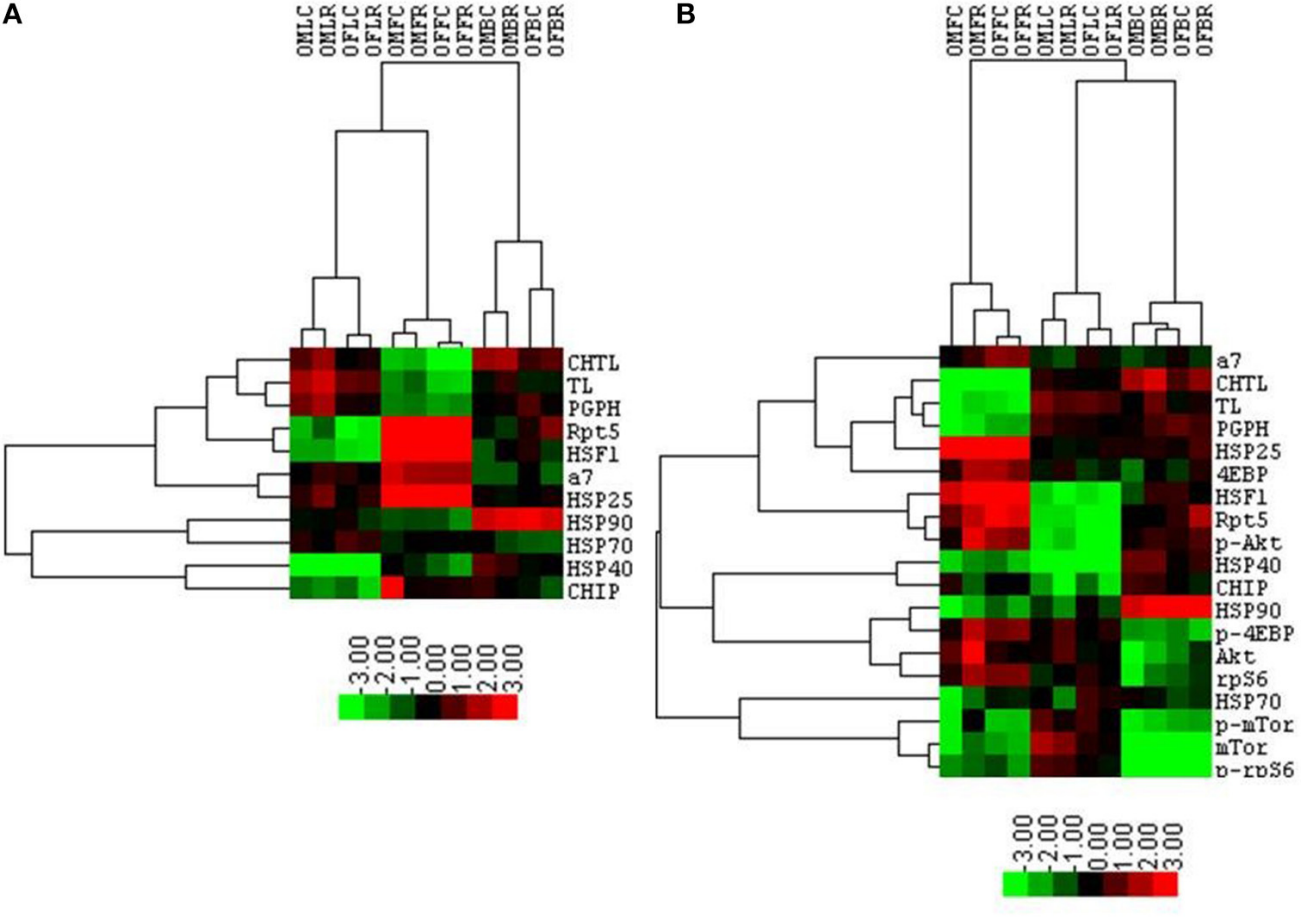
**Cluster analysis shows a proteasome-influenced grouping by tissue, sex, and then treatment**. A heat map showing results of the cluster analysis of variables including log-transformed peptidolytic activities and quantities of proteins determined by immuno-blot first with proteasome activity, proteasome subunits, and several proteasome-related chaperones together **(A)**, and then with the addition selected mTOR pathway proteins **(B)**. Groups representing the various lysates collected from animals treated with rapamycin and corresponding controls are organized in columns represent cases. Four letter codes define the groups with the first two in the code representing gender (OM, Old Male; OF, Old Female). The third letter represents the tissue (L, Liver; F, Fat; B, Brain), and the forth letter denotes treatment group (C, Control (Eudragit); R, Rapamycin-treated; i.e., OMFC, Old Male Fat Control). The three proteasome activity and protein tests are organized in rows. Results obtained with Cluster 3.0 analysis are shown. Heat maps were prepared with TreeView v.1.16r2. Color scheme corresponds to the normalized values of variables where bright green represent the lowest (approaching −3.0) and bright red the highest (close to +3.0) values. The lengths of tree branches are proportional to a relative similarity between variables and between cases.

Table 1 summarizes the observed rapamycin-mediated changes in proteasome activity, chaperones, and mTOR pathway proteins tested in this study. To determine whether the combined proteasome activities, chaperone, and mTOR pathway data assembled into common patterns of rapamycin-associated changes, cluster analyses were performed (Figure 11). First, proteasome activity and proteasome-related chaperones were clustered (Figure 11A). Cluster analysis revealed that groups separated by tissue type, weighted toward the higher levels of proteasome activity in liver and brain. These groups were further divided in the analysis by sex, but treatment was indistinguishable (Figure 11A). In this comparison, two distinct clusters formed. The first major cluster consisted of proteasome activity, the 19S ATPase RPT5 and HSF1in one sub-cluster with α7 and HSP25 in another. The second major cluster showed that the large chaperones (HSP90 and HSP70) and the HSP70 co-chaperones HSP40 and CHIP sub-divided into sub-clusters (Figure 11A).

The mTOR pathway proteins were added into the next cluster analysis (Figure 11B). This revealed that rapamycin-treated female brain and treated male liver formed two distinct groups. Three clusters formed in this analysis, with the mTOR pathway proteins 4EBP1 and p-AKT joining the “proteasome activity cluster” with the same proteins associated in the first Chaperone-Proteasome analysis from panel A (RPT5, α7, HSP25, and HSF1). A second cluster containing HSP90 and the co-chaperones HSP40 and CHIP also contained p-4EBP1, AKT, and rpS6 (Figure 11B). The last cluster in this analysis was characterized by low p-mTOR and low p-rpS6 (brain), and associated with HSP70 and total mTOR (Figure 11B).

## Discussion

In this study we evaluated if rapamycin-mediated changes in mTOR signaling and the ubiquitin proteasome system (i.e., proteasome activity and content and associated protein levels various chaperones) correlated with the sexually dimorphic effects of rapamycin on lifespan. Rapamycin-induced changes in proteasome activity and expression of the various molecular chaperones and mTOR pathway proteins were both sex- and tissue-specific (Figure 11). Rapamycin effects are readily apparent from both individual variables and cluster analyses (Figure 11). The latter were generated by applying multiple peptidolytic assays and associated chaperone proteins as well as a sampling of mTOR pathway proteins across the three tissues harvested from the same individuals. Cluster analyses reveal there are interactions between the proteasome-chaperone network and the mTOR pathway. Rapamycin treatment led to elevated proteasome activities in the brain of females, potentially promoting the removal of oxidatively damaged and misfolded proteins and creating improved proteostasis in female mice that are likely to contribute to the better protection of their brains against environmentally mediated (e.g., oxidatively damaged/ glucose crosslinking) protein aggregation. Better protection of the female brain may be an evolutionary life-history tradeoff and these traits may possibly vary with reproductive status (non-reproductive pregnant, lactation). Improved proteostasis and concomitant neuroprotection in the brains of rapamycin treated females supports previous studies using genetically engineered mouse models of Alzheimer's disease in which rapamycin firstly improved cognition and when subjected to a chronic high sugar diet prevented the accrual of protein aggregates, plaques and neurofibrillary tangles (Orr et al., 2014).

### Rapamycin effects are tissue-specific

Contrary to a previous data that examined only the 20S catalytic core ChTL activity (Zhang et al., 2014a), our extensive study revealed that rapamycin treatment did not globally suppress the PMDS or its associated chaperones. Indeed in our current study, declines in proteasome were observed predominantly in visceral fat (Figures 1, 4) in contrast the previous study using mice in the same cohort showed decreases in both heart and liver 20S ChTL proteasome activity in both sexes of rapamycin-treated mice (Zhang et al., 2014a). The decline in proteasome activity in peripheral tissues both in this study and that of Zhang, is in keeping with *in vitro* findings that rapamycin can allosterically inhibit proteasome activity (Osmulski and Gaczynska, 2013) However, this cannot explain the tissue specific increases in ChTL and TL proteasome activity in the brains of rapamycin treated animals.

While ChTL is considered the pivotal peptidolytic activity of oxidatively damaged proteins, other peptidase activities such as TL and PGPH activity may be more sensitive to rapamycin. Female mice treated with rapamycin showed the most divergent responses compared to untreated females for both the ChTL and TL catalytic sites in fat (decrease) and brain (increase) (Figure 1). PGPH proteasome activity did not change suggesting that rapamycin-mediated effects target specific types of proteins and peptides for cleavage driving changes in proteasome function rather than a restructuring of the proteasome although there is some evidence that mTOR signaling can induce proteasome subunits through both complex 1 or complex 2 (Lamming et al., 2014; Zhang et al., 2014b).

Given that rapamycin is likely to first reach the liver via the hepatic portal vein and be at its most concentrated there, it is surprising that of the three tissues examined, the liver was least sensitive to rapamycin treatment as shown by the peptide assays (Figure 1). It is possible that rapamycin is metabolized in such a way in the liver that it has minimal effects on liver proteasome function. Alternately, as the liver has several pathways involving nutrient signaling, a slight perturbation in the mTOR pathway is likely to be compensated for by other signaling pathways. Unfortunately, published RNA-sequence analyses data revealing differentially expressed genes in the transcriptome with rapamycin were equivocal (Fok et al., 2014) and did not reveal an obvious explanation for a lack of a change in 26S proteasome activity or a decline only in 26S TL activity as was seen in males with treatment (Figure 1). However, the in-gel assay on native gels did show a decline ChTL activity in lysates from rapamycin-treated samples from females (Figure 3) and a decline in α7 proteasome content suggesting alterations in liver proteasome expression and quantity by rapamycin(Zhang et al., 2014a). Given the low blood titer of C57BL6 compared to the UM-HET3 mice (~3–4 ng/L compared to 13.4 ng/L, respectively, after 6 months of treatment) (Harrison et al., 2009; Zhang et al., 2014a), we may have only observed effects in tissues that are particularly sensitive to rapamycin. Also, there definitely seem to be strain-dependent differences in at least rapamycin uptake or metabolism which could dictate the effect of the drug on measured parameters.

### Rapamycin decreases proteasome activity in visceral fat

We chose to include fat tissue in our analyses because we hypothesized it may be more sensitive to alterations in mTOR signaling, being a nutrient storage tissue, and was previously shown to be responsive male and female mice (Harrison et al., 2009). In C57BL6 mice, proteasome activity declines with age in adipose tissue (Dasuri et al., 2011). Our study shows that in old animals, there is a further reduction in proteolytic activity in visceral fat harvested from female mice after rapamycin-treatment (Figure 1) which was supported by a similar observation on native gel zymograms (Figure 4). This was accompanied by a treatment-related reduction in the key proteasome subunits α7 and RPT5 (Figures 4, 5). Thus, rapamycin appears to affect upstream control of critical genes in the PMDS in visceral fat though at this time it is unknown whether this effect is transcriptional or translational. Optimal function of the 26S is dependent on a tightly regulated ratio of ATPases such as RPT5 in the 19S regulatory cap (Smith et al., 2011). Alternatively, or in combination with an upstream regulation of RPT5 and/ or other proteasome ATPases, rapamycyin has been shown to directly block proteasome activity *in vitro* by interfering with the attachment of the 19S cap (Osmulski and Gaczynska, 2013). A similar mechanism may be in place in fat tissue whereby the inhibition of mTOR signaling in adipose tissue may block the binding of the regulatory cap to the catalytic core and impair proteasome function. Reduction of the proteasome activity has been shown to induce cytotoxicity, upregulate cell stress responses, and lead to various pathologies in other tissues (Goldbaum et al., 2006; Grimm et al., 2012). Taken together these data suggest that rapamycin-induced declines in visceral fat PMDS function could be detrimental and contribute to some of the observed peripheral tissue pathologies associated with age (Dasuri et al., 2011). For example, the development of obesity and insulin signaling in type 2 diabetes is influenced by the PMDS as insulin receptor substrate 1 is inactivated by degradation through this system (Sun et al., 1999; Chang et al., 2009). Thus, effects that mimic aging in PMDS function could substantially contribute to age-related insulin resistance in adipose tissue and other deleterious features associated with both fat and liver metabolism (Umemura et al., 2014). While we did not measure changes in insulin signaling in this cohort of animals, others have shown consistently that rapamycin affects the insulin pathway in much the same manner in multiple strains of mice including C57BL6, namely creating glucose intolerance, but causing insulin sensitivity (Lamming et al., 2013; Orr et al., 2014; Yu et al., 2014). PMDS could be a potential pathway to explain this phenomenon.

### Rapamycin increases proteasome activity in the brain

The most pronounced effects to proteasome activity were evident in the brain suggesting that it is not the rapamycin directly inducing these changes but rather the down-stream signaling it induces in rapamycin-responsive tissues. In contrast to the potential detrimental effects of rapamycin seen in visceral fat proteolytic function, the increase of PMDS in the brain could be beneficial and could suggest that organisms selectively protect certain more vulnerable tissues. Here, we observe that rapamycin induced an increase in proteasome activity, a phenotype commonly observed in long-lived species in the periphery as well as in the brain (Chondrogianni et al., 2000; Rodriguez et al., 2012; Edrey et al., 2014). Brain lysates from treated females showed both an increase in ChTL and TL activity (Figures 1, 2) as well as significant increases in 26S proteasome content on non-denaturing gels (Figure 2). Proteasome-related chaperones HSP70, C-terminal HSP-interacting protein (CHIP), RPT5, and HSP25 also showed significant increases in rapamycin-treated samples compared to control (Figure 5). Further, α7, RPT5,HSF1, and HSP25correlated strongly with higher levels of proteasome activity (Figure 11) in the cluster analysis. Other studies examining brains from rapamycin-treated mice have seen a similar enhancement of protein homeostasis and/or the HSP system (Spilman et al., 2010; Pierce et al., 2013; Orr et al., 2014). For example, rapamycin-fed mice showed the enhanced expression of the small chaperone gene, alpha-crystallin B chain (*CRYAB*) in the brains of a mouse Alzheimer's model (Pierce et al., 2013). Further, crossing this model with an HSF1-transgenic mouse (Pierce et al., 2010), which showed increased HSF1, HSP90, and CryAB, reduced toxic brain amyloid-β levels and improved cognitive function (Pierce et al., 2013). Interestingly, we did not see an increase in HSF1 in brain tissue with rapamycin treatment though cluster analyses suggested a correlation (Figures 5, 11). However, several chaperones showed increases in rapamycin-treated female brain samples (i.e., HSP70, CHIP, HSP25) suggesting both HSF1 and non-canonical upregulation of HSPs. One possibility is that if rapamycin indeed inhibits proteasome activity *in vivo* as suggested by *in vitro* studies (Osmulski and Gaczynska, 2013) an alternate heat shock factor such as HSF2 may be triggered inducing the same set of chaperones as HSF1 (Mathew et al., 1998).

The differences seen between the effects of rapamycin on the brain and peripheral tissues also suggests a tissue-specific decoupling of rapamycin function. In a study in which mice genetically mutated to serve as transgenic mouse models for Alzheimer's disease a tissue-specific decoupling of rapamycin effects were observed when these mice were fed a high sucrose diet and developed insulin resistance. While the exacerbation of plaques were ameliorated by the simultaneous treatment with rapamycin, rapamycin had no effect on peripheral insulin resistance and liver protein levels (Orr et al., 2014).

In this study, we did not examine markers of autophagy. Counter-intuitively, a previous investigation reports that, autophagy in brain tissue lysates was not increased in rapamycin-treated non-transgenic mice but only was manifest in animals genetically engineered to express high levels of amyloid-β or tau (Spilman et al., 2010; Orr et al., 2014). So while autophagy may have a key role in reducing aggregates from disease pathologies, non-aggregated protein degradation may instead be enhanced by the PMDS. The increase in chaperone-E3 ligase CHIP in the brain and a significant decline in liver tissue (Figure 5) may hold a clue. CHIP has been shown to be essential in modulating oxidative load and degradation of oxidized proteins through the PMDS as well as act as the E3 ligase for the degradation of tau, parkin, and polyglutamine expansions in brain tissue (Dickey et al., 2007; Sisoula and Gonos, 2011). As such, in the brain chronic rapamycin treatment could trigger an E3 ligase like CHIP to protect against proteotoxic stress, increasing the translation of chaperone proteins and the proteasome-mediated, protein degradation machinery to maintain protein equilibrium.

### Changes in mTOR signaling correlate with sex and tissue differences

In both female brain and fat tissues the mTOR pathway proteins, as expected, showed the most changes with rapamycin-treatment (Figures 3, 7; Table 1). However, while the changes in female fat samples suggest rapamycin blocks mTOR signaling, rapamycin-mediated changes in the brain did not (Figures 6, 9; Table 1) (reviewed in Wullschleger et al., 2006). Rather, these data suggest that mTOR remains active in the brain, and that the inhibitory effects of rapamycin are suppressed in brain tissue. This uncoupling of brain and peripheral effects on mTOR was unexpected but could also explain the lack of increased levels of autophagy in the brain of rapamycin-treated control animals seen in previous studies (Spilman et al., 2010; Orr et al., 2014). Further, the increase in p-rpS6 in the female brain (Figure 6) was very different to what was observed in female fat (Figure 9) or in the intestine of rapamycin-treated familial adenomatous polyposis mice both which showed a decrease in the phosphorylation state (ratio of phosphorylated to non-phosphorylated protein) of rp-S6 and other mTOR signaling molecules (Hasty et al., 2014). This paradox may be linked to the increase in both total and phosphorylated AKT (Figure 6, Table 1). A similar induced activation of AKT leading to a resistance of rapamycin treatment has been observed in human lung cancer cells and human rhabdomyosarcoma cell lines and in rodent cells overexpressing insulin-like growth factor (IGF) II (Sun et al., 2005; Wan et al., 2007). Unlike these cell systems, whereby the phosphorylation of both downstream mTOR targets S6K1 and 4EBP1 were suppressed, this was not observed in brain lysates from rapamycin-treated old females in this study (Figure 6). Instead we observed an increase in phosphorylated ribosomal protein S6, the target of S6K1 (Figure 6), responsible for 5′ terminal oligopyrimidine (TOP)-dependent translation (Wullschleger et al., 2006). Further, there was a decline in the phosphorylation state of 4EBP1 (Figure 6) which is responsible for control of cap-dependent translation (Wullschleger et al., 2006) and inhibition of TOP-dependent translation (Thoreen et al., 2012). This may be also be reflected by the cluster analysis correlation of low 4EBP1 with proteasome activity (Figure 11). These changes in translational control suggest a focus on TOP-dependent translation, and have further repercussions on the PMDS, as several proteasome-related HSP mRNAs can be preferentially translated through TOP-dependent translation during stress (Cuesta et al., 2000; Pierce et al., 2013). Conversely, in fat we observed a decline in phosphorylated rp-S6 and an increase in the phosphorylation state of 4EBP1 suggesting a decrease in translation (Figure 9) (Thoreen et al., 2012) that could in turn influence the observed reduction of proteasome activity (Figure 1).

Phosphorylation of AKT also protects against the apoptotic effects of proteasome inhibition further linking PMDS to the AKT cell survival program (Yu et al., 2006; Zanotto-Filho et al., 2012). As total AKT is also induced by 17-β estradiol (Haynes et al., 2000), this may explain why rapamycin-treated female animals have more robust effects when treated by the drug. Taken together, the sex-specific increase seen in brains of rapamycin treated mice in our comprehensive measures of proteasome activity could be linked to a sex-dependent change in AKT signaling driven by mTOR regulation of the heat-shock pathway through activated rp-s6 (as we observed in treated female brains; Figure 3). Interestingly, mTOR signaling through rp-S6 has also been shown to increase proteasome subunits with a dependence on nuclear factor erythroid-derived 2-related factor 1 (NRF1) (Zhang et al., 2014b). NRF1 also mediates the recovery of proteasome activity after stress or proteotoxic insults (Radhakrishnan et al., 2010; Balasubramanian et al., 2012).

## Conclusions

**Figure 12:**
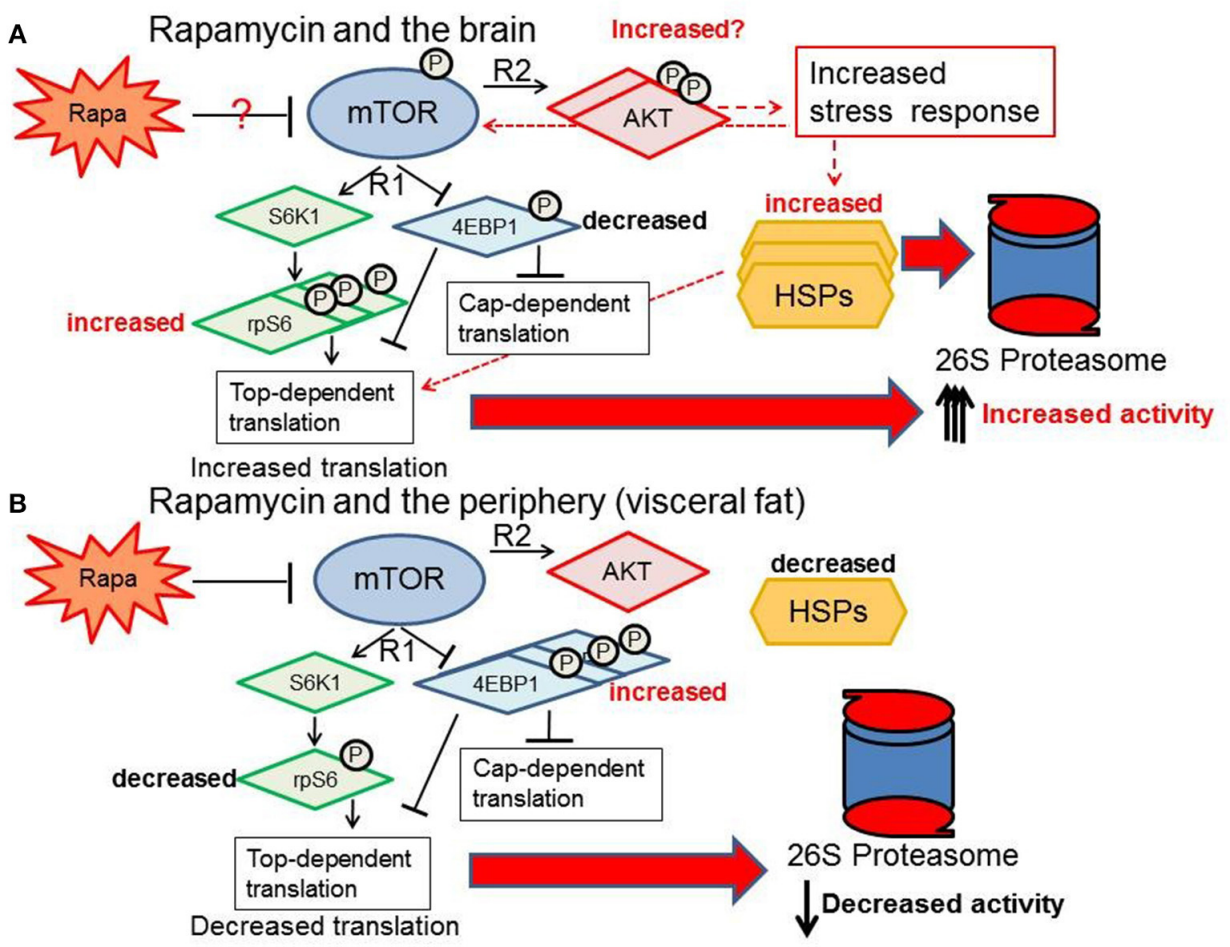
**Response of the proteasome-chaperone network differs between the brain and periphery. (A)** Rapamycin treatment may stimulate AKT in the brain, increasing proteasome-related chaperones through rp-S6 top-dependent translation and a non-cannonical stress response pathway (red-dashed arrows) ultimately increasing proteasome activity in the female brain. **(B)** In contrast, visceral fat shows a suppression of the mTOR network, reduction of the HSF1 chaperone response, and a decrease in proteasome activity.

Our data indicate that the sexually dimorphic effects of lifespan extension induced by rapamycin (Harrison et al., 2009; Fok et al., 2014; Miller et al., 2014; Zhang et al., 2014a) may be linked to sex differences in tissue-specific responsiveness of the regulators and components of mTOR, HSPs and PMDS pathways. Proteolytic activity is augmented in the brain facilitating more efficient removal of damaged or unfolded proteins, and as a consequence thereof, improved brain structural and functional integrity. This protective response to rapamycin treatment in brain tissue is uncoupled from that of the response in peripheral fat tissue. The latter, appears to be left more vulnerable to the potentially detrimental peripheral proteotoxic effects of rapamycin (Wilkinson et al., 2012; Ponticelli, 2014; Zhang et al., 2014a). A proposed summary of what happens to the PMDS in female brain vs. the periphery (specifically visceral fat) with rapamycin treatment is shown in Figure 12. Here we show that rapamycin appears to influence up- stream regulators of the proteasome-chaperone network through stimulation of the AKT pathway (Figure 12). Defining how the AKT pathway is stimulated or repressed in various tissues in a sex-dependent manner can give us insight on how to better understand rapamycin's effects in a therapeutic context. Upstream regulators of the PMDS including E3 ligases such as CHIP such as HSF1, 2 or NRF1 could be activated or suppressed by the AKT stress response in a sex-dependent manner thereby differentially affecting proteostasis in peripheral and brain tissues. Maintenance of protein homeostasis in the female brain may play a pivotal role in the extended longevity observed only in the rapamycin-treated female C57BL6 mice.

## Author contributions

Karl A. Rodriguez and Sherry G. Dodds conducted the experiments. Karl A. Rodriguez and Rochelle Buffenstein wrote the initial draft of the paper. Randy Strong, Veronica Galvan, Z. D. Sharp, and Rochelle Buffenstein contributed materials for the study. All authors helped with editing and revising the manuscript.

### Conflict of interest statement

None of the contributing authors received payment or services from a third party for any aspect of this submission. Z. D. Sharp, Randy Strong, and Veronica Galvan are uncompensated scientific advisors for Rapamycin Holdings, Inc. Z. D. Sharp, Randy Strong, and Veronica Galvan are part holders of a patent (#13/128/800 pending) for the use of encapsulated rapamycin in treating or preventing an age-related disease, condition, or disorder. There are no other relationships that could have influenced or give the appearance of potentially influencing this work. The authors declare that the research was conducted in the absence of any commercial or financial relationships that could be construed as a potential conflict of interest.

## References

[B1] Babbitt S. E. Kiss A. Deffenbaugh A. E. Chang Y. H. Bailly E. Erdjument-Bromage H.. (2005). ATP hydrolysis-dependent disassembly of the 26S proteasome is part of the catalytic cycle. Cell 121, 553–565. 10.1016/j.cell.2005.03.028 15907469

[B2] Balasubramanian S. Kanade S. Han B. Eckert R. L. (2012). A proteasome inhibitor-stimulated Nrf1 protein-dependent compensatory increase in proteasome subunit gene expression reduces polycomb group protein level. J. Biol. Chem. 287, 36179–36189. 10.1074/jbc.M112.359281 22932898 PMC3476285

[B3] Bonelli M. A. Desenzani S. Cavallini G. Donati A. Romani A. A. Bergamini E.. (2008). Low-level caloric restriction rescues proteasome activity and Hsc70 level in liver of aged rats. Biogerontology 9, 1–10. 10.1007/s10522-007-9111-9 17902036

[B4] Bucciantini M. Giannoni E. Chiti F. Baroni F. Formigli L. Zurdo J.. (2002). Inherent toxicity of aggregates implies a common mechanism for protein misfolding diseases. Nature 416, 507–511. 10.1038/416507a 11932737

[B5] Chang T. L. Chang C. J. Lee W. Y. Lin M. N. Huang Y. W. Fan K. (2009). The roles of ubiquitin and 26S proteasome in human obesity. Metabolism 58, 1643–1648. 10.1016/j.metabol.2009.05.020 19616267

[B6] Chondrogianni N. Petropoulos I. Franceschi C. Friguet B. Gonos E. S. (2000). Fibroblast cultures from healthy centenarians have an active proteasome. Exp. Gerontol. 35, 721–728. 10.1016/S0531-5565(00)00137-6 11053662

[B7] Chou S. D. Prince T. Gong J. Calderwood S. K. (2012). mTOR is essential for the proteotoxic stress response, HSF1 activation and heat shock protein synthesis. PLoS ONE 7:e39679. 10.1371/journal.pone.0039679 22768106 PMC3387249

[B8] Cuesta R. Laroia G. Schneider R. J. (2000). Chaperone hsp27 inhibits translation during heat shock by binding eIF4G and facilitating dissociation of cap-initiation complexes. Genes Dev. 14, 1460–1470. 10.1101/gad.14.12.1460 10859165 PMC316692

[B9] Dasuri K. Zhang L. Ebenezer P. Fernandez-Kim S. O. Bruce-Keller A. J. Szweda L. I.. (2011). Proteasome alterations during adipose differentiation and aging: links to impaired adipocyte differentiation and development of oxidative stress. Free Radic. Biol. Med. 51, 1727–1735. 10.1016/j.freeradbiomed.2011.08.001 21871954 PMC3378646

[B10] David D. C. Ollikainen N. Trinidad J. C. Cary M. P. Burlingame A. L. Kenyon C. (2010). Widespread protein aggregation as an inherent part of aging in C. elegans. PLoS Biol. 8:e1000450. 10.1371/journal.pbio.1000450 20711477 PMC2919420

[B11] De Hoon M. (2002). Cluster 3.0 Manual. Available Online at: http://bonsai.hgc.jp/~mdehoon/software/cluster/manual/Contents.html#Contents: Univeristy of Tokyo (Accessed November 5, 2002).

[B12] Demartino G. N. Gillette T. G. (2007). Proteasomes: machines for all reasons. Cell 129, 659–662. 10.1016/j.cell.2007.05.007 17512401

[B13] Dickey C. A. Patterson C. Dickson D. Petrucelli L. (2007). Brain CHIP: removing the culprits in neurodegenerative disease. Trends Mol. Med. 13, 32–38. 10.1016/j.molmed.2006.11.003 17127096

[B14] Edrey Y. H. Oddo S. Cornelius C. Caccamo A. Calabrese V. Buffenstein R. (2014). Oxidative damage and amyloid-beta metabolism in brain regions of the longest-lived rodents. J. Neurosci. Res. 92, 195–205. 10.1002/jnr.23320 24273049 PMC4838185

[B15] Elasser S. Schmidt M. Finley D. (2005). Characterization of the proteasome using native gel electrophoresis. Meth. Enzymol. 398, 353–363. 10.1016/S0076-6879(05)98029-4 16275342

[B16] Ferrington D. A. Husom A. D. Thompson L. V. (2005). Altered proteasome structure, function, and oxidation in aged muscle. FASEB J. 19, 644–646. 10.1096/fj.04-2578fje 15677694

[B17] Fok W. C. Chen Y. Bokov A. Zhang Y. Salmon A. B. Diaz V.. (2014). Mice fed rapamycin have an increase in lifespan associated with major changes in the liver transcriptome. PLoS ONE 9:e83988. 10.1371/journal.pone.0083988 24409289 PMC3883653

[B18] Goldbaum O. Riedel M. Stahnke T. Richter-Landsberg C. (2009). The small heat shock protein HSP25 protects astrocytes against stress induced by proteasomal inhibition. Glia 57, 1566–1577. 10.1002/glia.20870 19330846

[B19] Goldbaum O. Vollmer G. Richter-Landsberg C. (2006). Proteasome inhibition by MG-132 induces apoptotic cell death and mitochondrial dysfunction in cultured rat brain oligodendrocytes but not in astrocytes. Glia 53, 891–901. 10.1002/glia.20348 16609961

[B20] Grimm S. Hohn A. Grune T. (2012). Oxidative protein damage and the proteasome. Amino Acids 42, 23–38. 10.1007/s00726-010-0646-8 20556625

[B21] Grune T. Catalgol B. Licht A. Ermak G. Pickering A. M. Ngo J. K.. (2011). HSP70 mediates dissociation and reassociation of the 26S proteasome during adaptation to oxidative stress. Free Radic. Biol. Med. 51, 1355–1364. 10.1016/j.freeradbiomed.2011.06.015 21767633 PMC3172204

[B22] Harrison D. E. Strong R. Sharp Z. D. Nelson J. F. Astle C. M. Flurkey K.. (2009). Rapamycin fed late in life extends lifespan in genetically heterogeneous mice. Nature 460, 392–395. 10.1038/nature08221 19587680 PMC2786175

[B23] Hasty P. Livi C. B. Dodds S. G. Jones D. Strong R. Javors M.. (2014). eRapa restores a normal life span in a FAP mouse model. Cancer Prev. Res. 7, 169–178. 10.1158/1940-6207.CAPR-13-0299 24282255 PMC4058993

[B24] Haynes M. P. Sinha D. Russell K. S. Collinge M. Fulton D. Morales-Ruiz M.. (2000). Membrane estrogen receptor engagement activates endothelial nitric oxide synthase via the PI3-kinase-Akt pathway in human endothelial cells. Circ. Res. 87, 677–682. 10.1161/01.RES.87.8.677 11029403

[B25] Kruegel U. Robison B. Dange T. Kahlert G. Delaney J. R. Kotireddy S.. (2011). Elevated proteasome capacity extends replicative lifespan in Saccharomyces cerevisiae. PLoS Genet. 7:e1002253. 10.1371/journal.pgen.1002253 21931558 PMC3169524

[B26] Lamming D. W. Demirkan G. Boylan J. M. Mihaylova M. M. Peng T. Ferreira J.. (2014). Hepatic signaling by the mechanistic target of rapamycin complex 2 (mTORC2). FASEB J. 28, 300–315. 10.1096/fj.13-237743 24072782 PMC3868844

[B27] Lamming D. W. Ye L. Astle C. M. Baur J. A. Sabatini D. M. Harrison D. E. (2013). Young and old genetically heterogeneous HET3 mice on a rapamycin diet are glucose intolerant but insulin sensitive. Aging Cell 12, 712–718. 10.1111/acel.12097 23648089 PMC3727050

[B28] Liu C.-W. Li X. Thompson D. Wooding K. Chang T.-I. Tang Z.. (2006). ATP Binding and ATP hydrolysis play distinct roles in the function of 26S Proteasome. Mol. Cell 24, 39–50. 10.1016/j.molcel.2006.08.025 17018291 PMC3951175

[B29] Livingstone M. Atas E. Meller A. Sonenberg N. (2010). Mechanisms governing the control of mRNA translation. Phys. Biol. 7:021001. 10.1088/1478-3975/7/2/021001 20463379

[B30] Massey A. C. Kiffin R. Cuervo A. M. (2006). Autophagic defects in aging - Looking for an emergency exit? Cell Cycle 5, 1292–1296. 10.4161/cc.5.12.2865 16760669

[B31] Mathew A. Mathur S. K. Morimoto R. I. (1998). Heat shock response and protein degradation: regulation of HSF2 by the ubiquitin-proteasome pathway. Mol. Cell Biol. 18, 5091–5098. 9710593 10.1128/mcb.18.9.5091 PMC109094

[B32] Miller R. A. Harrison D. E. Astle C. M. Baur J. A. Boyd A. R. De Cabo R.. (2011). Rapamycin, but not resveratrol or simvastatin, extends life span of genetically heterogeneous mice. J. Gerontol. A Biol. Sci. Med. Sci. 66, 191–201. 10.1093/gerona/glq178 20974732 PMC3021372

[B33] Miller R. A. Harrison D. E. Astle C. M. Fernandez E. Flurkey K. Han M.. (2014). Rapamycin-mediated lifespan increase in mice is dose and sex dependent and metabolically distinct from dietary restriction. Aging Cell 13, 468–477. 10.1111/acel.12194 24341993 PMC4032600

[B34] Orr M. E. Salinas A. Buffenstein R. Oddo S. (2014). Mammalian target of rapamycin hyperactivity mediates the detrimental effects of a high sucrose diet on Alzheimer's disease pathology. Neurobiol. Aging 35, 1233–1242. 10.1016/j.neurobiolaging.2013.12.006 24411482 PMC3973159

[B35] Osmulski P. A. Gaczynska M. (2013). Rapamycin allosterically inhibits the proteasome. Mol. Pharmacol. 84, 104–113. 10.1124/mol.112.083873 23619386

[B36] Pierce A. Podlutskaya N. Halloran J. J. Hussong S. A. Lin P. Y. Burbank R.. (2013). Over-expression of heat shock factor 1 phenocopies the effect of chronic inhibition of TOR by rapamycin and is sufficient to ameliorate Alzheimer's-like deficits in mice modeling the disease. J. Neurochem. 124, 880–893. 10.1111/jnc.12080 23121022 PMC6762020

[B37] Pierce A. Wei R. Halade D. Yoo S. E. Ran Q. Richardson A. (2010). A Novel mouse model of enhanced proteostasis: full-length human heat shock factor 1 transgenic mice. Biochem. Biophys. Res. Commun. 402, 59–65. 10.1016/j.bbrc.2010.09.111 20920476

[B38] Ponticelli C. (2014). The pros and the cons of mTOR inhibitors in kidney transplantation. Exp. Rev. Clin. Immunol. 10, 295–305. 10.1586/1744666X.2014.872562 24377908

[B39] Radhakrishnan S. K. Lee C. S. Young P. Beskow A. Chan J. Y. Deshaies R. J. (2010). Transcription factor Nrf1 mediates the proteasome recovery pathway after proteasome inhibition in mammalian cells. Mol. Cell 38, 17–28. 10.1016/j.molcel.2010.02.029 20385086 PMC2874685

[B40] Rodriguez K. A. Edrey Y. H. Osmulski P. Gaczynska M. Buffenstein R. (2012). Altered composition of liver proteasome assemblies contributes to enhanced proteasome activity in the exceptionally long-lived naked mole-rat. PLoS ONE 7:e35890. 10.1371/journal.pone.0035890 22567116 PMC3342291

[B41] Rodriguez K. A. Gaczynska M. Osmulski P. A. (2010). Molecular mechanisms of proteasome plasticity in aging. Mech. Ageing Dev. 131, 144–155. 10.1016/j.mad.2010.01.002 20080121 PMC2849732

[B42] Rodriguez K. A. Osmulski P. Pierce A. Weintraub S. T. Gaczynska M. Buffenstein R. (2014). A cytosolic protein factor from the naked mole-rat activates proteasomes of other species and protects these from inhibition. Biochim. Biophys. Acta. 1842, 2060–2072. 10.1016/j.bbadis.2014.07.005 25018089 PMC4829350

[B43] Ross C. A. Poirier M. A. (2004). Protein aggregation and neurodegenerative disease. Nat. Med. 10(Suppl.). S10–S17. 10.1038/nm1066 15272267

[B44] Sisoula C. Gonos E. S. (2011). CHIP E3 ligase regulates mammalian senescence by modulating the levels of oxidized proteins. Mech. Ageing Dev. 132, 269–272. 10.1016/j.mad.2011.04.003 21510971

[B45] Smith D. M. Fraga H. Reis C. Kafri G. Goldberg A. L. (2011). ATP binds to proteasomal ATPases in pairs with distinct functional effects, implying an ordered reaction cycle. Cell 144, 526–538. 10.1016/j.cell.2011.02.005 21335235 PMC3063399

[B46] Spilman P. Podlutskaya N. Hart M. J. Debnath J. Gorostiza O. Bredesen D.. (2010). Inhibition of mTOR by rapamycin abolishes cognitive deficits and reduces amyloid-beta levels in a mouse model of Alzheimer's disease. PLoS ONE 5:e9979. 10.1371/journal.pone.0009979 20376313 PMC2848616

[B47] Sun S. Y. Rosenberg L. M. Wang X. Zhou Z. Yue P. Fu H.. (2005). Activation of Akt and eIF4E survival pathways by rapamycin-mediated mammalian target of rapamycin inhibition. Cancer Res. 65, 7052–7058. 10.1158/0008-5472.CAN-05-0917 16103051

[B48] Sun X. J. Goldberg J. L. Qiao L. Y. Mitchell J. J. (1999). Insulin-induced insulin receptor substrate-1 degradation is mediated by the proteasome degradation pathway. Diabetes 48, 1359–1364. 10.2337/diabetes.48.7.1359 10389839

[B49] Tai H. C. Besche H. Goldberg A. L. Schuman E. M. (2010). Characterization of the brain 26S Proteasome and its interacting proteins. Front. Mol. Neurosci. 3:12. 10.3389/fnmol.2010.00012 20717473 PMC2901091

[B50] Thoreen C. C. Chantranupong L. Keys H. R. Wang T. Gray N. S. Sabatini D. M. (2012). A unifying model for mTORC1-mediated regulation of mRNA translation. Nature 485, 109–113. 10.1038/nature11083 22552098 PMC3347774

[B51] Umemura A. Park E. J. Taniguchi K. Lee J. H. Shalapour S. Valasek M. A.. (2014). Liver damage, inflammation, and enhanced tumorigenesis after persistent mTORC1 inhibition. Cell Metab. 20, 133–144. 10.1016/j.cmet.2014.05.001 24910242 PMC4079758

[B52] Vernace V. A. Arnaud L. Schmidt-Glenewinkel T. Figueiredo-Pereira M. E. (2007). Aging perturbs 26S proteasome assembly in Drosophila melanogaster. FASEB J. 21, 2672–2682. 10.1096/fj.06-6751com 17413001 PMC3435146

[B53] Wan X. Harkavy B. Shen N. Grohar P. Helman L. J. (2007). Rapamycin induces feedback activation of Akt signaling through an IGF-1R-dependent mechanism. Oncogene 26, 1932–1940. 10.1038/sj.onc.1209990 17001314

[B54] Wilkinson J. E. Burmeister L. Brooks S. V. Chan C. C. Friedline S. Harrison D. E.. (2012). Rapamycin slows aging in mice. Aging Cell 11, 675–682. 10.1111/j.1474-9726.2012.00832.x 22587563 PMC3434687

[B55] Wullschleger S. Loewith R. Hall M. N. (2006). TOR signaling in growth and metabolism. Cell 124, 471–484. 10.1016/j.cell.2006.01.016 16469695

[B56] Yeung K. Y. Ruzzo W. L. (2001). Principal component analysis for clustering gene expression data. Bioinformatics 17, 763–774. 10.1093/bioinformatics/17.9.763 11590094

[B57] Yu C. Friday B. B. Lai J. P. Yang L. Sarkaria J. Kay N. E.. (2006). Cytotoxic synergy between the multikinase inhibitor sorafenib and the proteasome inhibitor bortezomib *in vitro*: induction of apoptosis through Akt and c-Jun NH2-terminal kinase pathways. Mol. Cancer Ther. 5, 2378–2387. 10.1158/1535-7163.MCT-06-0235 16985072

[B58] Yu Z. Wang R. Fok W. C. Coles A. Salmon A. B. Perez V. I. (2014). Rapamycin and dietary restriction induce metabolically distinctive changes in mouse liver. J. Gerontol. A Biol. Sci. Med. Sci. [Epub ahead of print]. 10.1093/gerona/glu053 24755936 PMC4447794

[B59] Zanotto-Filho A. Braganhol E. Battastini A. M. Moreira J. C. (2012). Proteasome inhibitor MG132 induces selective apoptosis in glioblastoma cells through inhibition of PI3K/Akt and NFkappaB pathways, mitochondrial dysfunction, and activation of p38-JNK1/2 signaling. Invest. New Drugs 30, 2252–2262. 10.1007/s10637-012-9804-z 22367315

[B60] Zhang Y. Bokov A. Gelfond J. Soto V. Ikeno Y. Hubbard G.. (2014a). Rapamycin extends life and health in C57BL/6 mice. J. Gerontol. A Biol. Sci. Med. Sci. 69, 119–130. 10.1093/gerona/glt056 23682161 PMC4038246

[B61] Zhang Y. Nicholatos J. Dreier J. R. Ricoult S. J. Widenmaier S. B. Hotamisligil G. S.. (2014b). Coordinated regulation of protein synthesis and degradation by mTORC1. Nature 513, 440–443. 10.1038/nature13492 25043031 PMC4402229

